# Application of cyclic phosphonamide reagents in the total synthesis of natural products and biologically active molecules

**DOI:** 10.3762/bjoc.10.195

**Published:** 2014-08-13

**Authors:** Thilo Focken, Stephen Hanessian

**Affiliations:** 1 Xenon Pharmaceuticals Inc., 3650 Gilmore Way, Burnaby, BC V5G 4W8, Canada; 2 Department of Chemistry, Université de Montréal, C.P. 6128, Succursale Centre-Ville, Montréal, QC H3C 3J7, Canada

**Keywords:** conjugate addition, cyclopropanation, olefination, organophosphorus, phosphonamide, total synthesis

## Abstract

A review of the synthesis of natural products and bioactive compounds adopting phosphonamide anion technology is presented highlighting the utility of phosphonamide reagents in stereocontrolled bond-forming reactions. Methodologies utilizing phosphonamide anions in asymmetric alkylations, Michael additions, olefinations, and cyclopropanations will be summarized, as well as an overview of the synthesis of the employed phosphonamide reagents.

## Introduction

**Figure 1:**
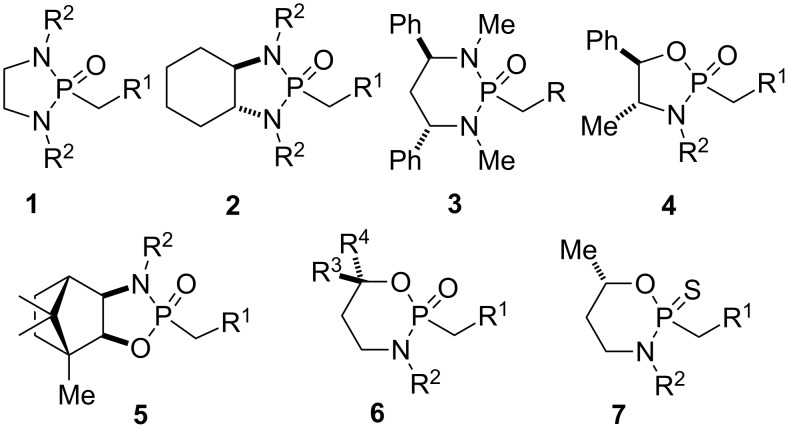
Examples of phosphonamide reagents used in stereoselective synthesis.

Chiral non-racemic and achiral cyclic phosphonamide reagents **1–7** (Figure 1) have been employed in organic synthesis primarily as stabilized anionic nucleophiles in addition reactions to electrophilic substrates with good to excellent stereocontrol. The products obtained from these reactions were used as key building blocks in the total synthesis of a variety of structurally highly diverse and complex natural products and also of biologically active compounds.

The phosphonamide anions are derived from a small number of common motifs as shown in Figure 1. Diazaphospholidine **2** was introduced by Hanessian and co-workers, and represents the most commonly used phosphonamide in organic synthetic transformations [1]. This chiral phosphonamide typically yields reaction products with excellent stereocontrol, which are easily isolated as diastereometrically pure or highly enriched compounds. Many are crystalline solids that can be purified further by recrystallization. Diazaphosphorinane **3** and oxazaphosphorinanes **6** and **7** have been extensively studied by Denmark and co-workers [2–4]. Oxazaphospholidine **4** was independently developed by Hua [5–6], Steglich [7] and their respective co-workers. Camphor oxazaphospholidine **5** was reported by Sisti and co-workers [8–9].

**Figure 2:**
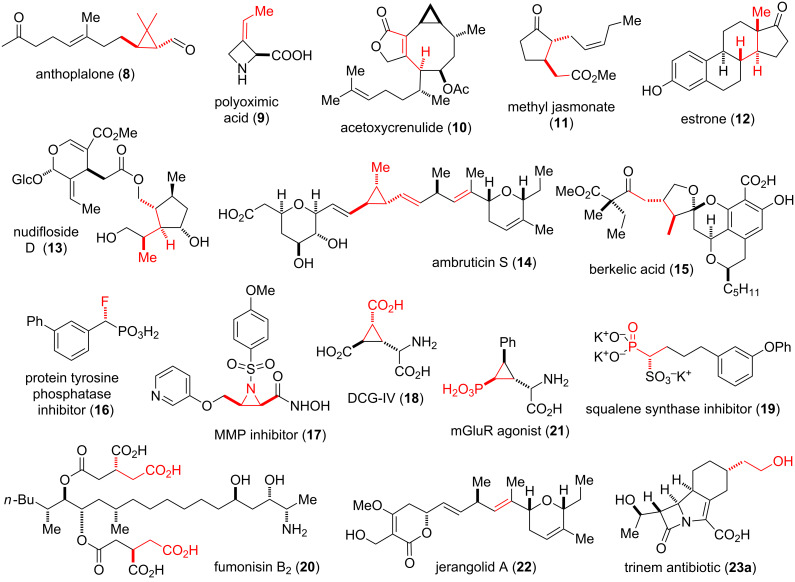
Natural products and bioactive molecules synthesized using phosphonamide-based chemistry (atoms, bonds and stereocenters formed by phosphonamide reagents are highlighted in red).

This review focuses on the application of phosphonamide reagents in the total synthesis of natural products and biologically active compounds. An overview of the molecules synthesized by phosphonamide technology is shown in Figure 2. Each molecule shown will be discussed in detail later in this review. First, relevant methodologies utilizing phosphonamides will be discussed, followed by an overview of synthetic routes for the preparation of phosphonamide reagents.

## Review

### Phosphonamides in stereoselective synthesis

For the purpose of this review, only phosphonamide methodologies with applications in the synthesis of natural products or bioactive molecules will be discussed [10]. Similar technologies to the ones discussed here without such applications and other uses of chiral phosphonamide reagents in asymmetric synthesis such as Denmark’s carbanion-accelerated Claisen rearrangements [11–12] have been reviewed elsewhere [13–14]. These methodologies will be mentioned where appropriate but not discussed in detail.

#### Olefination

**Scheme 1:**
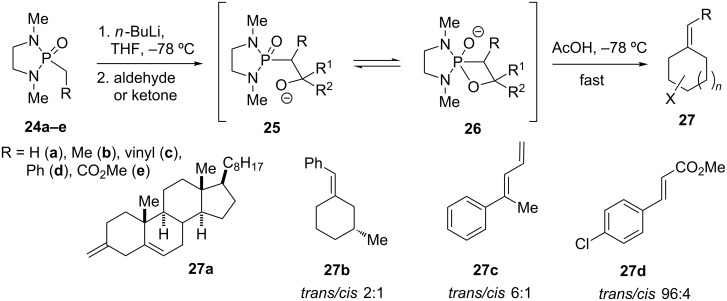
Olefination with cyclic phosphonamide anions, mechanistic rationale, and selected examples **27a–d** [18].

Monocyclic phosphonamide reagents of type **1** bearing a *N*,*N*’-dialkylethane-1,2-diamine backbone were first reported as olefination reagents by Corey and Cane [15] and later by Savignac [16–17], and Hanessian [18] and their co-workers. Deprotonation of phosphonamides of type **1** affords weakly basic anions which are excellent reagents for the transformation of aldehydes and ketones into the corresponding alkenes **27** via intermediates **25** and **26** (Scheme 1) [18]. Contrary to their acyclic *N*,*N*-diethyl phosphonamides, which require harsher conditions to undergo fragmentation [19], cyclic oxaphosphetane oxide **26** releases the corresponding olefin **27** upon treatment with cold acetic acid [18]. Moreover, the only weakly basic nature of the “soft” phosphonamide anion favors the attack on the carbonyl group over enolization. Thus, treatment of Δ5-cholestenone with **24a** yielded the unconjugated olefin **27a** in addition to recovered unreacted enone, whereas phosphorus ylides would form Δ4-cholestenone via enolization and double bond conjugation [20].

Other monocyclic phosphonamides with application in olefination reactions are those derived from 1-(*tert*-butylamino)-2-methylpropan-2-ol. Denmark and Amburgey used this type of phosphonamides in a four-step protocol for the highly stereoselective synthesis of trisubstituted alkenes [21].

**Scheme 2:**
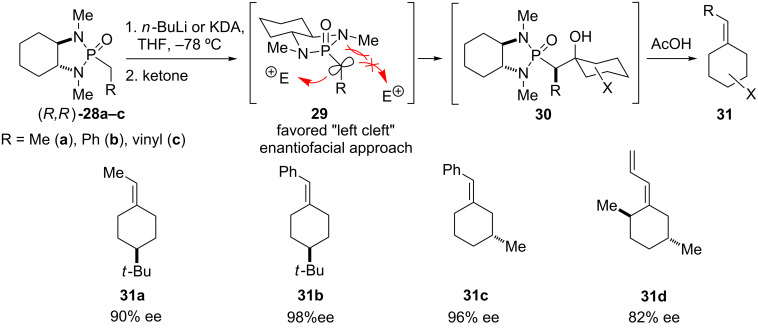
Asymmetric olefination with chiral phosphonamide anions and selected examples **31a–d** [1,22].

The application of cyclic phosphonamides was further extended toward asymmetric olefination reactions by Hanessian and co-workers, using a chiral, non-racemic diamine to generate the corresponding olefination reagents [1,22–23]. To this end the *C_2_*-symmetric phosphonamide (*R*,*R)*-**28** derived from *trans*-(*R*,*R*)-*N*,*N’*-dimethyl-1,2-diaminocyclohexane [14] was conceived of as a chiral version of *N,N*’-dialkylethane-1,2-diamine phosphonamide **24** (Scheme 1 and Scheme 2). The reaction of anions **29** with ketones leads to the corresponding β-hydroxy phosphonamide intermediates **30**, which undergo elimination of the intermediate oxaphosphetanes to give chiral olefins **31** with moderate to high enantioselectivities. The attack of electrophiles E is favored from the “left cleft” of the anion **29** in the (*R*,*R*)-isomer due to steric and stereoelectronic effects. Intermediates such as **30** can be isolated and purified as crystalline solids suitable for X-ray analysis if water is used for quenching instead of acetic acid.

Olefinations based on phosphonamides were employed in the construction of di- and trisubstituted double bonds in the total synthesis of polyoximic acid [24–26], jerangolid A [27], and ambruticin S [28], as discussed later in this review.

#### Alkylations and aminations

**Scheme 3:**
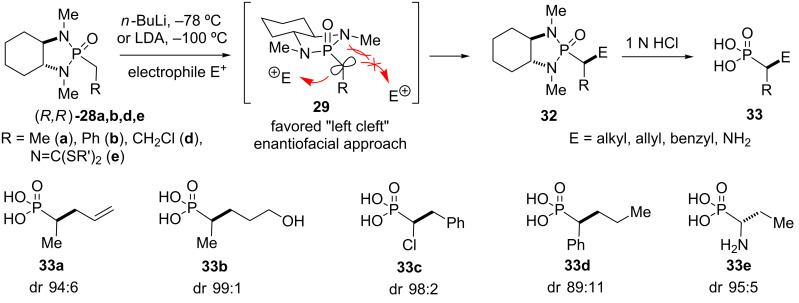
Synthesis of α-substituted phosphonic acids **33a–e** by asymmetric alkylation of chiral phosphonamide anions, mechanistic rationale, and selected examples after hydrolysis. Diastereomeric ratios (dr) refer to the corresponding phosphonamide adducts **32** before hydrolysis [22–23].

Treatment of α-phosphoryl carbanions with alkyl halides gives stereoselective access to a variety of α-substituted alkylphosphonic acids (Scheme 3). The attack on the alkyl halide occurs from the “left cleft” side of the anion **29** in the (*R*,*R*)-isomer. Thus, α-substituted phosphonamides **32** can be obtained in good to excellent diastereoselectivity and further hydrolyzed to the corresponding enantiomerically pure α-substituted phosphonic acids **33** [22,29–30]. Other asymmetric alkylation methodologies using chiral phosphonamides were reported by the groups of Denmark [3–4] and Steglich [7].

Remarkably, the alkylation of α-dithioalkylimino phosphonamide **28e** provided a diastereomer of **32** with the opposite configuration of the newly formed stereocenter. This inversion in asymmetric induction relative to non-heteroatom substituted phosphonamides such as **28a** is presumably a result of a chelated intermediate that exposes the opposite face of the anion to the electrophile compared to the conventionally accepted model [31]. The use of other electrophiles for the stereoselective formation of C–N bonds has also been reported. Thus, α-amino-α-alkyl phosphonic acids [32–34] could be obtained through amination and azidation of phosphonamide anions, respectively, and subsequent conversion of the primary adducts [35]. The enantioselective synthesis of α-phosphonosulfonic acids as squalene inhibitors, as discussed later in this review, was achieved using similar reactions – asymmetric alkylation of an α-sulfo phosphonamide and asymmetric α-sulfuration of an α-alkyl phosphonamide, respectively [36].

#### Michael reactions

**Scheme 4:**
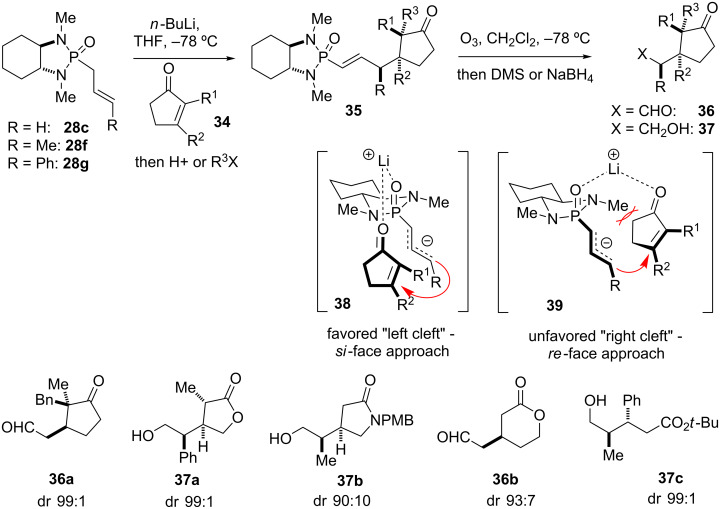
Asymmetric conjugate additions of *C_2_*-symmetric chiral phosphonamide anions to cyclic enones, lactones, lactams, and α,β-unsaturated esters with optional enolate alkylations; mechanistic rationale, and selected examples **36** and **37** after cleavage of the auxiliary. Diastereomeric ratios (dr) refer to the corresponding phosphonamide adducts **35** before ozonolysis–reduction [37–40].

The application of chiral, cyclic phosphonamides such as **28c** in asymmetric Michael-type reactions has proven to be a powerful tool in natural product synthesis to generate up to three contiguous stereogenic centers in a single step with a high level of stereocontrol (Scheme 4) [37–40]. Thus, vicinal and quartenary carbon centers can be obtained in high diasteromeric purity by conjugate additions of allyl, crotyl, and cinnamyl-derived anions to Michael acceptors such as enones, lactones, lactams, and α,β-unsaturated esters followed by optional alkylation to give adducts **35**. The stereoselectivity of the reaction can be explained by lithium-coordinated intermediate **38**, in which chelated Michael acceptors are best accommodated within the “left-cleft” of the (*R*,*R*)-reagents **28c** and **28f**,**g**. The resulting vinyl phosphonamide product bearing the chiral auxiliary can be cleaved by ozonolysis to the corresponding aldehydes **36** and the latter reduced to alcohols **37**, respectively, as shown in Scheme 4. Many highly functionalized, vicinally substituted compounds could be prepared by this method in good to excellent enantiopurity [37–40].

**Scheme 5:**
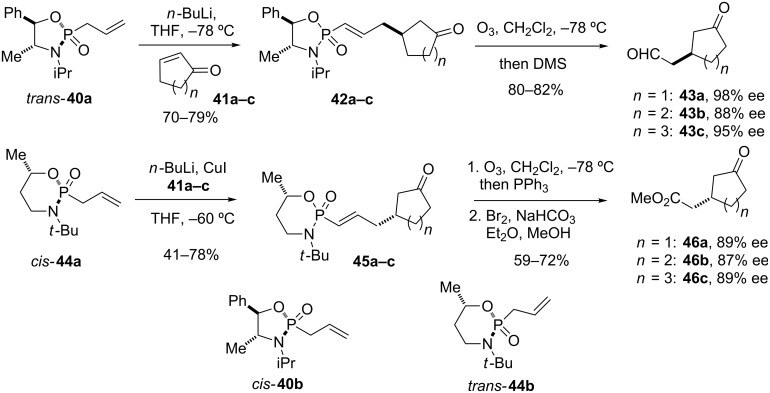
Asymmetric conjugate additions of *P*-chiral phosphonamide anions generated from **40a** and **44a** to cyclic enones, and further derivatization of the adducts [2–6].

Asymmetric conjugate additions using *P*-chiral phosphonamides were reported by Denmark [2–4] and Hua [5–6], with remarkable differences in selectivity depending on the configuration of the *P*-stereogenic center (Scheme 5). Thus, the addition of the Li-anion of *trans*-**40a** to cyclic enones **41** proceeded with a high level of stereocontrol, providing adducts **42** with up to 98% ee. *Cis* and *trans* refer to the orientation of the *P*-alkyl group relative to the *N*-alkyl group, in agreement with Denmark’s naming of oxazaphosphorinanes [2–4]. Thus, *trans* describes a compound with a *S*-configurated phosphorus center, whereas *cis* confers to a *R*-configuration. Degradation of the adducts by ozonolysis yielded oxocycloalkane-3-carboxaldehydes **43**, which are useful synthetic intermediates. Diastereomer *cis*-**40b** however gave only poor diastereofacial selection, providing the corresponding 1,4-addition adducts in 28–64% ee [5]. In a similar fashion, Denmark’s oxazaphosphorinane *cis*-**44a** yielded keto esters **46a–c** in high optical purities via conjugate addition to enones **41** followed by ozonolysis and oxidative esterification. Using diastereomer *trans*-**44b** on the other hand provided ketoesters **46a–c** with only 10–15% ee [2].

Asymmetric Michael additions using phosphonamides **28c**,**f**, or analogs of **28** and **40**, respectively, were applied in the total synthesis of acetoxycrenulide (**10**) [41–42], berkelic acid (**15**) [43], estrone (**12**) [44], fumonisin B_2_ (**20**) [45–47], methyl jasmonate (**11**) [48], and nudifloside A and D (**13**) [49], as discussed later in this review. Studies for the synthesis of the polyphenolic natural products tatanans A–C also explored the use of phosphonamide technology [50]. The discussion of the latter natural products is not included in this review, as phosphonamide technology was only used for limited exploratory studies.

#### Cyclopropanation and aziridination

**Scheme 6:**
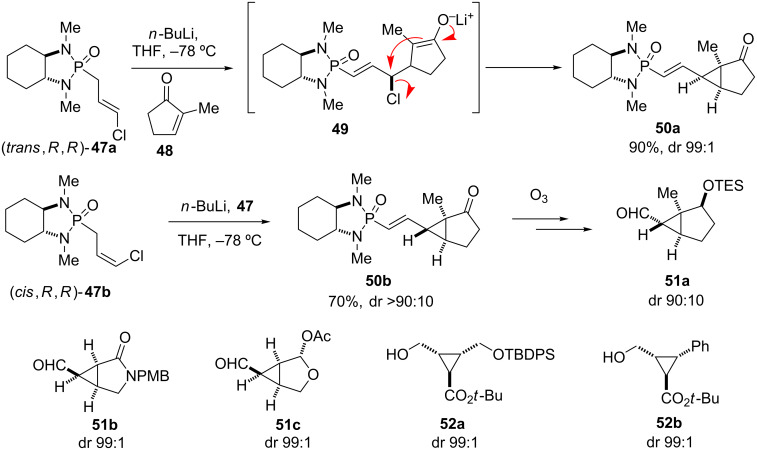
Asymmetric cyclopropanation with chiral chloroallyl phosphonamide **47**, mechanistic rationale, and selected examples **51** and **52** after cleavage of the auxiliary and further derivatization. Diastereomeric ratios (dr) refer to the corresponding phosphonamide adducts **50** before ozonolysis–reduction [51].

The cyclopropanation of α,β-unsaturated esters and lactones using chiral phosphonamide reagents is a special case of the conjugate addition–enolate alkylation sequence. The application of chloroallyl phosphonamides such as (*trans*,*R*,*R*)-**47a** in the conjugate addition to enones provides the corresponding fused *endo*,*endo*-cyclopropane **50a** in high diastereomeric excess [51]. The transformation proceeds via the intermediate Michael adduct **49**, which eliminates chloride after stereocontrolled attack of the enolate to afford cyclopropane **50a**. Starting with (*cis*,*R*,*R*)-**47b**, the isomeric *exo*,*endo* product **50b** is obtained as major isomer. The cyclopropanation reaction tolerates a wide range of Michael acceptor subtrates such as enones, lactones, lactams, and acyclic α,β-unsaturated esters. The obtained products can easily be cleaved to the corresponding aldehydes **51** by ozonolysis, reduced further to alcohols **52**, and constitute versatile cyclopropane chirons (Scheme 6) [51–55].

The cyclopropanation with chloroallyl phosphonamide **47a** was used to construct the cyclopropane fragments of anthoplalone (**8**) [56], ambruticin S (**14**) [28], and mGluR agonist DCG-IV (**18**) [57], as discussed later in this review. Studies for the synthesis of ottelione A and B [58] also employed this cyclopropanation methodology using a mixture of **47a** and **47b**. The discussion of the latter natural products is not included in this review, as phosphonamide technology was only used for limited exploratory studies.

**Scheme 7:**
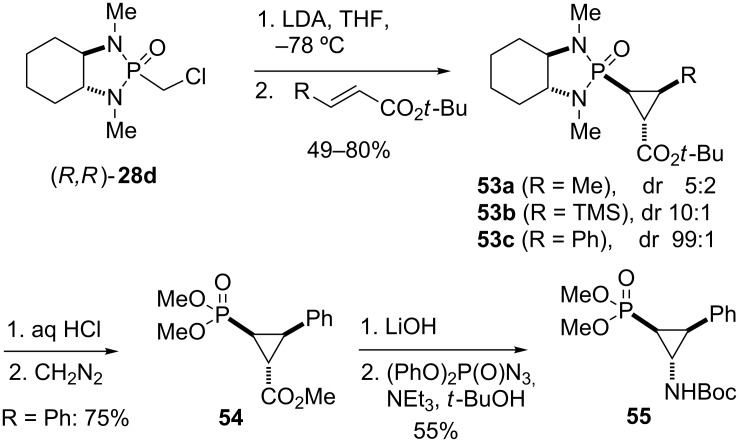
Asymmetric cyclopropanation with chiral chloromethyl phosphonamide **28d** [59].

Replacing chloroallyl phosphonamides **47** with chloromethyl phosphonamide **28d** in the addition to α,β-unsaturated esters also gives cyclopropane products, which can be converted to cyclopropylphosphonic acids **54** and aminocyclopropylphosphonic acids **55** (Scheme 7) [59]. The synthesis of an mGluR agonist was achieved using chloromethyl phosphonamide **28d** [60–61], as discussed later in this review.

**Scheme 8:**
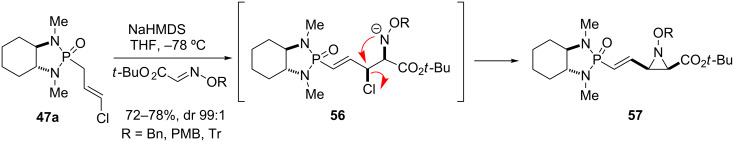
Stereoselective synthesis of *cis*-aziridines **57** from chiral chloroallyl phosphonamide **47a** [62].

Replacing Michael acceptors with oximes in the reaction with chloroallyl phosphonamide **47a** leads to the stereoselective formation of *cis*-aziridines **57** (Scheme 8) [62]. Thus, addition of the anion of phosphonamide **47a** to *tert*-butyl glyoxylate *O*-protected oximes affords the corresponding aziridine adducts **57** in excellent diastereoselectivity in a Darzens-type reaction via intermediate **56**. This aziridination methodology was then used in the synthesis of MMP-inhibitors [63], as discussed later in this review. Aziridines are also obtained as primary products in the addition of chloromethyl phosphonamide **28d** to imines. The initial attack leads to a α-chloro-β-amino phosphonamide adduct as intermediate, which then undergoes intramolecular cyclization to form the aziridine ring after elimination of chloride. When *N*-substituted aromatic imines are used, the corresponding aziridines can be reduced at the benzylic carbon to give α-aminophosphonic acids [64].

### Synthesis of phosphonamides

**Scheme 9:**
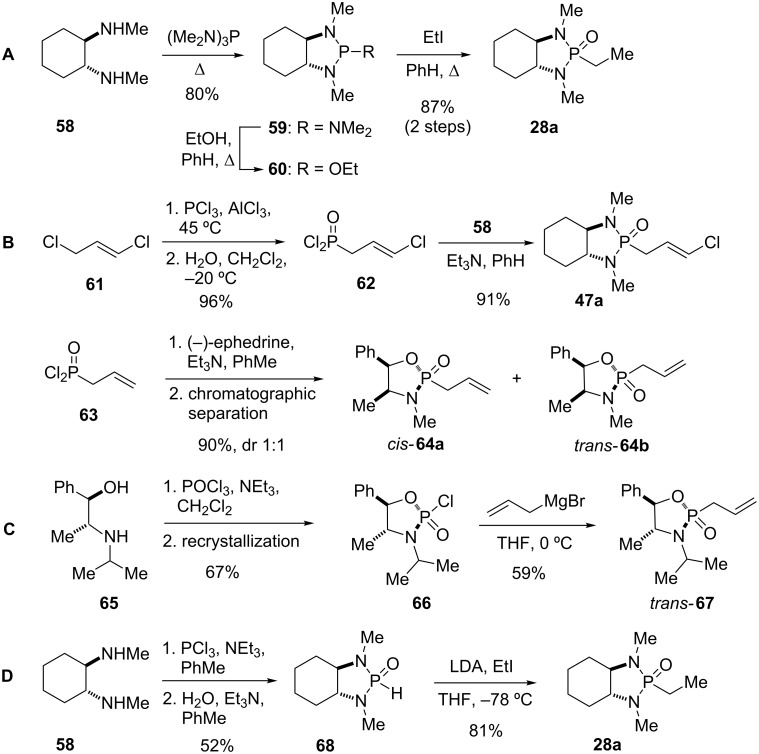
Synthesis of phosphonamides by (A) Arbuzov reaction, (B) condensation of diamines with phosphonic acid dichlorides, (C) nucleophilic displacement, (D) alkylation of phosphorus acid diamides [1,5–6,28,30,51,69].

There are four major methods for the synthesis of phosphonamides: (A) Arbuzov reaction, (B) condensation of diamines with phosphonic acid dichlorides, (C) nucleophilic displacement, and (D) alkylation of 2-oxo-1,3,2-diazaphospholidine (Scheme 9). All of these methods were employed to prepare the phosphonamide reagents used in the synthesis of the natural products and bioactive compounds discussed in this review.

#### Phosphonamides by Arbuzov reaction

An example for the application of the Arbuzov reaction is the synthesis of phosphonamide (*R*,*R*)-**28a**. Thus, heating of (*R,R*)*-N*,*N*’-dimethyl-1,2-diaminocyclohexane (**58**) with hexamethylphosphorus triamide gave the distillable phospholane **59**, which was further converted with ethanol into **60**. Treatment with ethyl iodide in an Arbuzov reaction provided the desired ethyl phosphonamide **28a** (Scheme 9A) [1,30]. Cyclic phosphonamides derived from *C_2_-*symmetric diamines such as **28a** do not have a stereogenic P-atom and therefore exist as a single pair of enantiomers. An example for the synthesis of a complex phosphonamide by the Arbuzov reaction can be found in the total synthesis of estrone (**12**) [44], as discussed later in this review.

#### Phosphonamides by condensation of diamines with phosphonic acid dichlorides

The most commonly applied method for the synthesis of simple phosphonamides is the condensation of phosphonic acid dichlorides with a diamine. Thus, treatment of acid dichloride **62** with *(R*,*R)-N*,*N*’-dimethyl-1,2-diaminocyclohexane (**58**) afforded cyclopropanation reagent **47a** [28,51]. The required phosphonic acid dichlorides can be obtained either from chlorination of phosphonic acids [65–66] or from treatment of an allyl chloride such as **61** with phosphorus trichloride followed by hydrolysis to give **62** [15,67]. Reactions of unsymmetrical amines or aminoalcohols such as ephedrine with phosphonic acid dichlorides result in the generation of a stereogenic center at the P-atom and thus to diastereomeric phosphonamides *cis-***64a** and *trans-***64b**, which typically can be separated by chromatography [5].

Cyclopropanation reagent **47a** was used in the total synthesis of anthoplalone (**8**) [56] and ambruticin S (**14**) [28], whereas an unsymmetrical phosphonamide of type **64** was used in the synthesis of PTP inhibitors [68], and methyl jasmonate [48], as discussed later in this review.

#### Nucleophilic displacement

The stereoselective synthesis of unsymmetrical phosphonamides **67** by nucleophilic displacement was reported by Hua and co-workers [6]. Treatment of aminoalcohol **65** with phosphoryl chloride provided **66** as a mixture of diastereomers (dr 93:7), from which pure **66** was obtained by recrystallization. Chloride displacement at phosphorus with allylmagnesium bromide proceeded with retention of configuration to give allyl phosphonamide **67**. A similar displacement reaction was used to generate a phosphonamide reagent in the synthesis of squalene synthase inhibitors [36] and is discussed later in this review.

#### Phosphonamides by alkylation of phosphorus acid diamides

Spilling and co-workers reported the preparation of alkyl phosphonamides through alkylation of bicyclic phosphite anions [69–71]. Thus, condensation of diamine **58** with phosphorus trichloride followed by hydrolysis of the formed 2-chloro-1,3,2-diazaphospholidine with one equivalent of water gave phosphorus acid diamide **68**. The latter could be deprotonated with LDA at low temperature and alkylated to give phosphonamide **28a**. Spilling’s alkylation methodology was used in the total synthesis of jerangolid A (**22**) [27] and ambruticin S (**14**) [28].

### Application in total synthesis

#### Polyoximic acid (1993)

**Figure 3:**
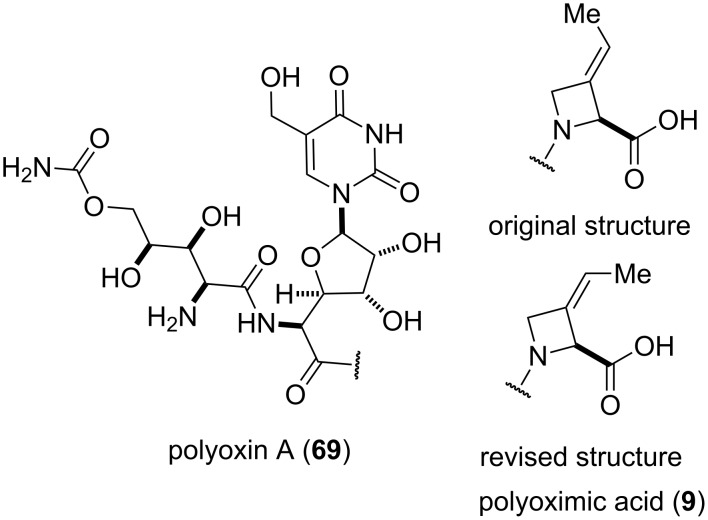
Original and revised structure of polyoxin A (**69**) [24–26].

Polyoximic acid (**9**) is a unique amino acid that occurs only as a component of polyoxins A, F, H, and K, which exhibit antibiotic properties [72]. Originally, the stereochemistry of the exocyclic double bond of polyoximic acid was incorrectly assigned as *E* based on a low resolution nOe experiment (Figure 3). The total synthesis of polyoximic acid (**9**) by Hanessian and co-workers led to a reassignment of its structure and that of the parent molecules, such as polyoxin A (**69**) [24–26].

**Scheme 10:**
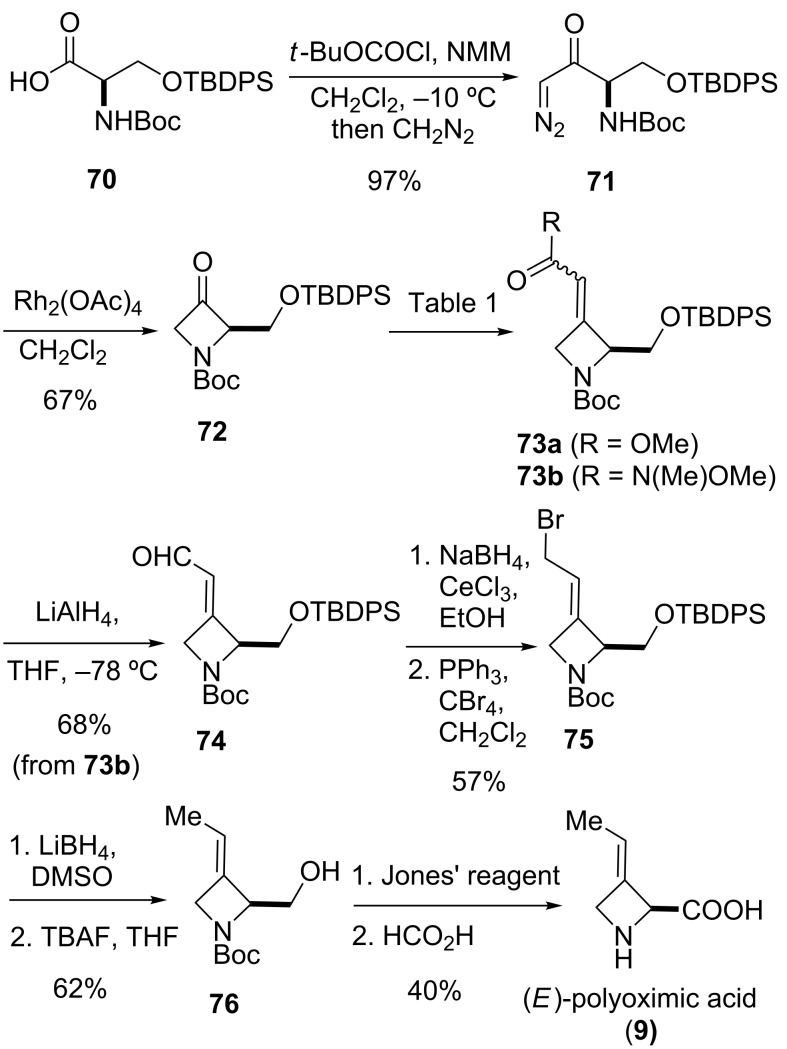
Synthesis of (*E*)-polyoximic acid (**9**) [24–26].

The synthesis of the *E*-isomer of polyoximic acid started from protected D-serine **70**, which was converted into diazoketone **71** by reacting a mixed anhydride with diazomethane (Scheme 10). Azetidinone **72** was then formed through a rhodium-catalyzed intramolecular carbenoid insertion into the N–H bond as the first pivotal step of the synthesis. The next key step was to introduce the exocyclic double bond with control of the stereochemistry of the double bond. For that purpose, a variety of ‘typical’ Wittig and Horner–Wadsworth–Emmons reagents were screened. In addition, cyclic phosphonamides were utilized as olefination reagents (Table 1).

**Table 1:** Horner–Wadsworth–Emmons olefination of ketone **72** [26].

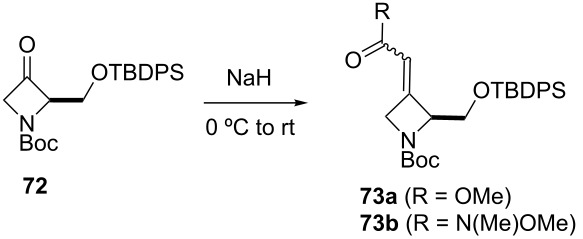					

Entry	Reagent	Solvent	Product	Yield (%)	*E*/*Z*

1	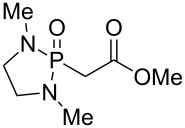 **24e**	THF	**73a**	71	80:20
2	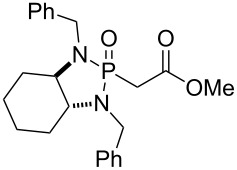 **77**	THF	**73a**	62	91:9
3	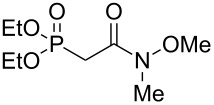 **78**	DME	**73b**	61	87:13
4	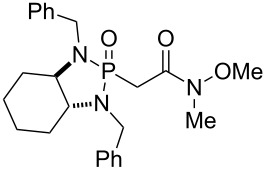 **79**	THF	**73b**	83	88:12
5^a^	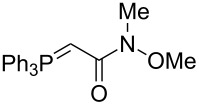 **80**	MeOH	**73b**	73	10:90

^a^Without NaH, at −78 °C.

Employing phosphonamides **24e** and **77** in the olefination of **72** favored the formation of the desired *E*-isomer of **73a**, however the mixture of isomers was inseparable by normal chromatographic methods. The chiral backbone of **77** had a beneficial effect on the stereoselectivity of the olefination, with an improved *E/Z* ratio of 91:9 compared to the achiral analogue **24e** (Table 1, entries 1 and 2). Employing phosphonate-Weinreb amide **78** and phosphonamide-Weinreb amide **79** not only afforded good *E*/*Z* ratios of 87:13 to 88:12 of **73b** but also provided a product that could now be separated by column chromatography (Table 1, entries 3 and 4). Reduction of amide *E*-**73b** by LiAlH_4_ to aldehyde **74** and further reduction under Luche conditions delivered an allylic alcohol, which was then converted into bromide **75**. Debromination and cleavage of the TBDPS protecting group gave protected amino-alcohol **76**. Finally, Jones oxidation and removal of the *N*-Boc protecting group produced crystalline (*E*)-polyoximic acid (*E*-**9**), whose structure was unambiguously confirmed by X-ray analysis. A comparison of the NMR spectrum of *E*-**9** with an authentic sample of natural polyoximic acid led to the conclusion that the natural product contains a *Z*-double bond, contrary to the original assignment (Figure 3).

The synthesis of *Z*-polyoximic acid (*Z*-**9**) was eventually achieved through a similar sequence as shown in Scheme 10. Replacing phosphonamide **79** with Wittig-reagent **80** as olefinating reagent gave a separable *E*/*Z* mixture in a ratio of 1:9 in favor of the desired *Z*-isomer of **73b** (Table 1, entry 5). The latter was then transformed into *Z*-**9** in an analogous fashion as described for the *E*-isomer. Comparison of the spectroscopic and physical data of synthetic *Z*-**9** with the amino acid derived from the natural product confirmed the revised structure of the latter [24–26,73].

#### Acetoxycrenulide (1995)

**Figure 4:**
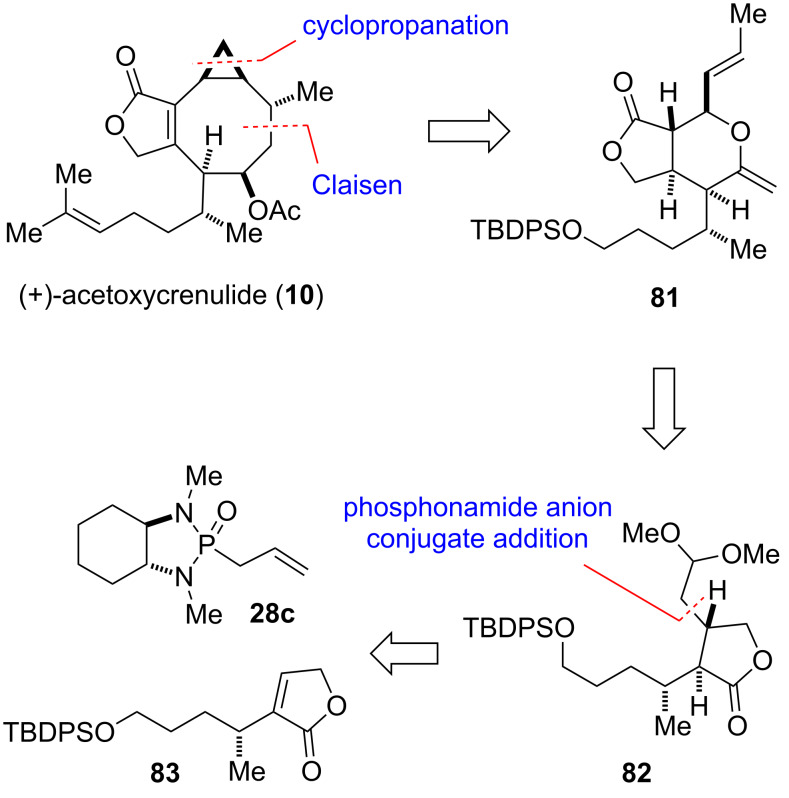
Key assembly strategy of acetoxycrenulide (**10**) [41–42].

The marine toxin acetoxycrenulide (**10**) was isolated independently from small brown seaweed of the family *Dictyotaceae* and from the sea hare [74–75]. Paquette and co-workers reported the first and only total synthesis of this diterpene (Figure 4) [41–42]. The cyclooctanoid core of the target was envisioned to be formed by a Claisen rearrangement of intermediate **81**. The latter and most of its stereocenters would originate from lactone **82**, which in turn is the product of a conjugate addition of chiral allyl phosphonamide reagent **28c** to butenolide **83** prepared from (*R*)-citronellol. The correct installation of the stereocenters of **82** was crucial to the success of the synthesis, as they would form a template for the stereocontrolled incorporation of the remaining stereocenters.

**Scheme 11:**
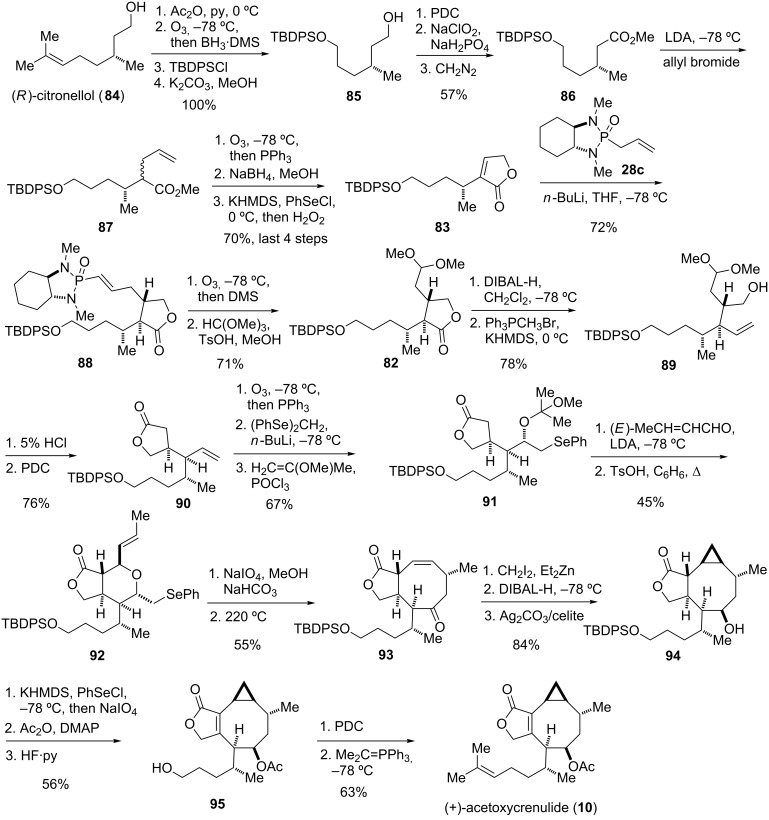
Total synthesis of (+)-acetoxycrenulide (**10**) [41–42].

The construction of butenolide **83** started from (*R*)-citronellol (**84**), which could in principle, deliver the entire alkenyl side chain of acetoxycrenulide (**10**) (Scheme 11). However, the double bond needed to be transformed into a TBDPS ether as it would not survive the late-stage cyclopropanation (Figure 4). Thus, protection of the primary alcohol as acetate, ozonolysis with reductive work-up, treatment with TBDPSCl and ensuing hydrolytic removal of the acetate yielded mono-protected diol **85**. Conversion into methyl ester **86** by a three-step procedure and subsequent alkylation with allyl bromide gave alkene **87**. Ozonolysis with reductive work-up was followed by spontaneous cyclization to the corresponding γ-lactone, which was then transformed into **83** by means of α-selenenylation, oxidation, and elimination (Scheme 11).

With butenolide **83** in hand the stage was set for one of the key steps of the synthesis. Addition of the anion of phosphonamide **28c** to **83** proceeded with a high level of facial and *cis*/*trans*-selectivity to afford adduct **88** as a single diastereomer with the correct stereochemistry. Removal of the chiral auxiliary by ozonolysis, protection of the resulting aldehyde, reduction of the lactone ring to the lactol, and treatment with methylenetriphenylphosphorane delivered **89**. Mild acidic hydrolysis of the acetal followed by oxidation then yielded **90** with the γ-lactone unit that constitutes ring A of acetoxycrenulide. Cleavage of the double bond by ozonolysis and addition of (phenylseleno)methyllithium followed by protection of the formed hydroxy group provided **91** as a single diastereomer. Condensation of **91** with (*E*)-crotonaldehyde and heating of the obtained aldol adduct with catalytic amount of acid formed tetrahydropyran **92** as key intermediate of the synthesis. Oxidation of **92** and heating to 220 ºC resulted in a concurrent selenoxide elimination and Claisen rearrangement to give **93** via intermediate **81**. Face-selective Simmons–Smith cyclopropanation, reduction of both carbonyl groups, and chemoselective oxidation of the formed lactol with Fetizon’s reagent afforded **94**. The final steps of the synthesis involved conversion to the corresponding α,β-unsaturated lactone **95** and modification of the side chain to re-build the original double bond to eventually give (+)-acetoxycrenulide (**10**) [41–42].

#### Squalene synthase inhibitor (1996)

**Scheme 12:**
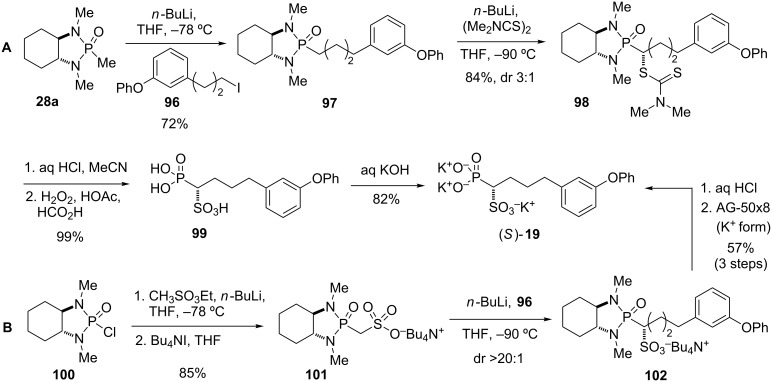
Synthesis squalene synthase inhibitor **19** by asymmetric sulfuration (A) and asymmetric alkylation (B) of a phosphonamide anion [36].

Inhibitors of squalene synthase have sparked interest as selective cholesterol lowering agents [76–77]. The enzyme is involved in the first committed step in the cholesterol synthesis and catalyses the conversion of two molecules of farnesyl diphosphate into squalene, which is later converted exclusively into various sterols, such as cholesterol, by a multi-step pathway [78]. The α-phosphono sulfonate **19** was found to be a potent inhibitor of squalene synthase, however, only the racemic version was originally tested. Biller and co-workers designed an enantioselective synthesis of **19** based on an asymmetric sulfuration (route A) or asymmetric alkylation (route B) of a chiral phosphorus carbanion (Scheme 12) [36]. Deprotonation of (*R*,*R*)-**28a** and alkylation with 3-(3’-phenoxyphenyl)propyl iodide (**96**) gave **97**. Sulfuration of the Li anion of **97** with tetramethylthiuram disulfide provided the adduct as a 3:1 mixture of diastereomers, with **98** as the major isomer. The low diastereoselectivivity observed for the sulfuration as compared to that reported for the alkylation of phosphonamides similar to **97** was explained with a longer C–S bond in the transition state and the steric hindrance sensed by a thiuram relative to an alkyl halide. Further support for this theory comes from a control experiment, in which **97** was alkylated under the same conditions with benzyl bromide, leading to a 10:1 mixture of diastereomers. The pure diastereomer **98** was then hydrolyzed with mild acid to remove the chiral auxiliary and oxidized to diacid **99**. Converison into its potassium salt yielded squalene synthase inhibitor (*S*)-**19**. In a similar sequence, the minor diastereomer from the sulfuration, 1-*epi* **98**, was converted into the opposite enantiomer (*R*)-**19** (Scheme 12A).

Reversing the steps for the introduction of the alkyl chain and the sulfonate moiety with the aim to achieve better selectivity led to route B (Scheme 12B). Thus, treatment of **100**, obtained from (*R*,*R*)-*N,N*’-dimethyl-1,2-diaminocyclohexane and phosphoryl chloride, with the anion generated from ethyl methanesulfonate followed by cleavage of the ethyl sulfonate gave tetrabutylammonium salt **101**. Deprotonation of **101** followed by reaction of the dianion with 3-(3’-phenoxyphenyl)propyl iodide (**96**) provided adduct **102** with excellent selectivity (dr >20:1). Removal of the chiral auxiliary and purification by cation exchange finally afforded (*S*)-**19**.

Both enantiomers of **19** were tested in *in vitro* assays for their ability to inhibit squalene synthase. Enantiomer (*S*)-**19** was found to be 16-fold more potent than the (*R*)-enantiomer, with IC_50_ values of 68 and 1120 nM, respectively [36].

#### Fumonisin B_2_ (1997)

**Figure 5:**
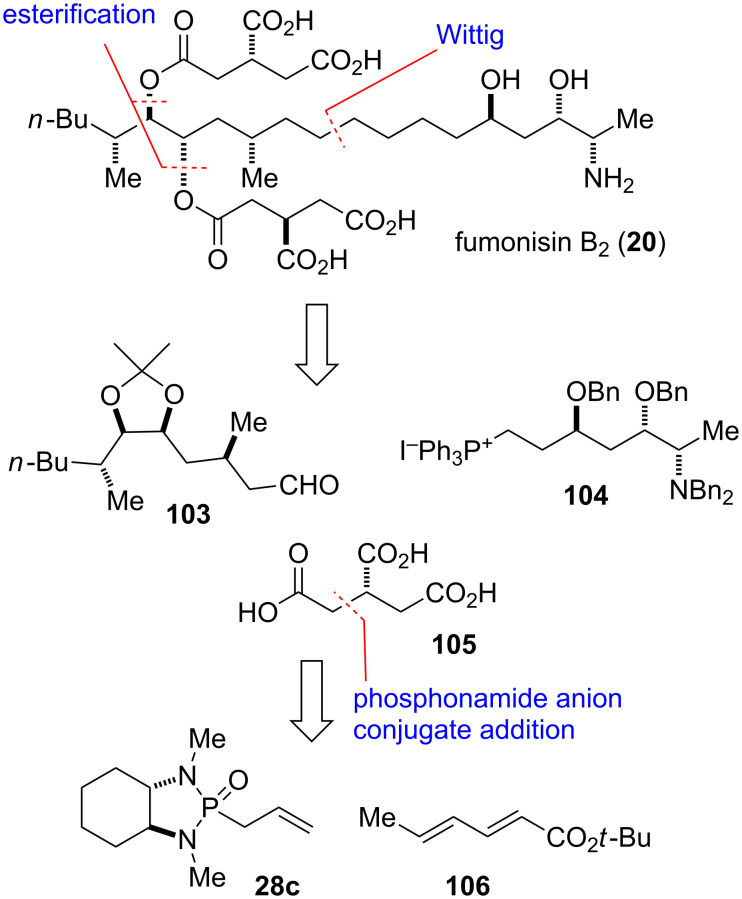
Key assembly strategy of fumonisin B_2_ (**20**) and its tricarballylic acid fragment **105** [45–46].

**Scheme 13:**
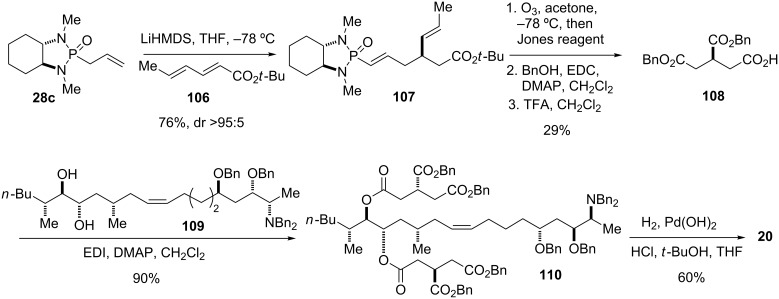
Final steps of the total synthesis of fumonisin B_2_ (**20**) [45–46].

Fumonisin B_2_ (**20**) belongs to the family of fumonisin mycotoxins produced by fungi of the genus *Fusarium*, a common grain mold. It is a close structural analogue of fumonisin B_1_, the most prevalent member of the family of fumonisins [79–80]. Fumonisin B_1_, B_2_ and other fumonisins frequently contaminate maize and other crops [81–83]. Kishi and co-workers adopted a convergent approach to fumonisin B_2_, with the molecule being cleaved into three main fragments **103–105** [45–46]. The connection of **103** and **104** under formation of the fumonisin backbone would employ a Wittig reaction, followed by attachment of two molecules of tricarballylic acid (**105**). The latter fragment would be accessed by conjugate addition of the anion of phosphonamide **28c** to *tert*-butyl sorbate (**106**) to give intermediate **107** followed by oxidative cleavage of the chiral auxiliary (Figure 5 and Scheme 13).

The preparation of **107** was performed as previously reported with minor modifications (Scheme 13) [37]. Thus, addition of the Li anion of **28c** to *tert*-butyl sorbate (**106**) afforded adduct **107** with excellent diastereoselectivity. Cleavage of both double bonds by ozonolysis followed by oxidative work-up with Jones’ reagent provided a monoprotected tricarballylic acid intermediate. Conversion of the free carboxylic acid moieties into their benzyl esters followed by cleavage of the *tert*-butyl ester gave **108**. This fragment was then coupled with diol **109** to afford the fully protected fumonisin B_2_ precursor **110**. Final hydrogenation and hydrogenolysis of all eight benzyl protecting groups was accomplished using Pearlman’s catalyst under mild acidic conditions to give fumonisin B_2_ (**20**) [45–47,84].

#### Tricylic β-lactams (1997)

**Figure 6:**
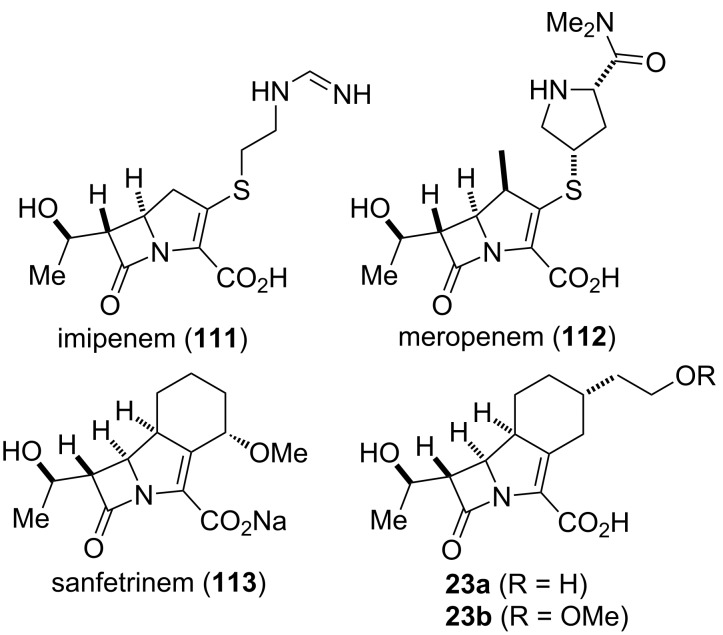
Selected examples of two subclasses of β-lactam antibiotics – carbapenems (**111** and **112**) and trinems (**113** and **23**).

β-Lactam antibiotics are the most prescribed and successful class of antibiotics developed and used in clinical practice. This broad class of antibiotics shares a highly reactive four-membered β-lactam ring and includes penicillin derivatives, cephalosporins, monobactams, carbapenems, and other related compounds [85–87]. Approved drugs such as imipenem (**111**) and meropenem (**112**) (Figure 6) belong to the subclass of carbapenems, which are powerful antibiotics with a broad spectrum of activity against Gram-positive and Gram-negative bacteria and are often used as antibiotics for many hard-to-treat bacterial infections, such as *Escherichia coli* and *Klebsiella pneumoniae* [88–89]. Resistance of bacterial strains to antibiotics has dictated the need for continuous development of existing and discovery of new antibiotics ever since the introduction of the first antibacterial agents in the first half of the 20^th^ century. In recent years, this trend has become a serious threat for public health with the emergence of carbapenem-resistant enterobacteriaceae such as *Klebsiella pneumoniae* [90–91].

In the 1990’s, scientists at GlaxoWellcome developed sanfetrinem (**113**), a member of a novel class of tricyclic β-lactam antibiotics known as trinems [92–93]. Eventually the development of sanfetrinem was stopped in 2009 after phase II clinical trials [86], but the compound inspired others to study its structural variants. Hanessian and co-workers reported on the synthesis of analogs of sanfetrinem (**113**) [94–96], including the 5α-hydroxyethyltrinems **23a**,**b** (Figure 6) [97], as well as a total synthesis of sanfetrinem (**113**) [96].

**Scheme 14:**
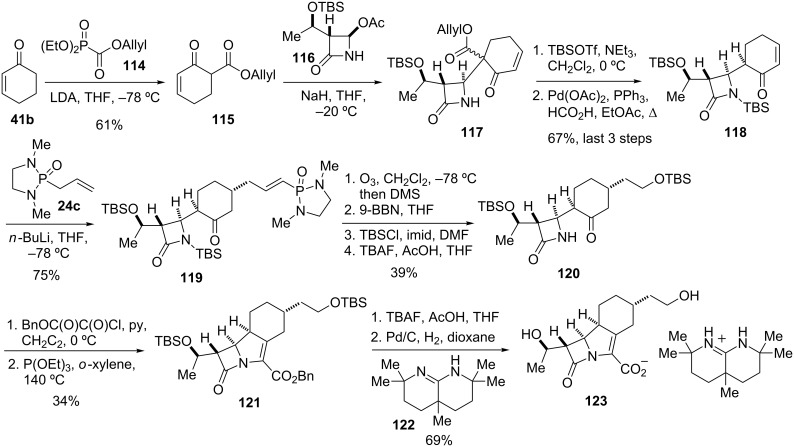
Synthesis of tricyclic β-lactam antibiotic **123** [97].

The installation of the two-carbon side chain of **23** was achieved through a stereoselective conjugate addition of a phosphonamide allyl anion to an advanced intermediate (Scheme 14). The latter was constructed in four steps starting from cyclohexenone (**41b**). Thus, addition of the Li salt of **41b** to allyl diethylphosphonoformate (**114**) afforded β-ketoester **115**, which in turn was condensed with commercially available azetidinone **116** to give **117** as a mixture of diastereomers. Protection of the nitrogen with TBS triflate followed by deprotection of the allyl carboxylate with formic acid under palladium catalysis and subsequent decarboxylation yielded enone **118** as a single diastereomer.

Addition of the Li anion of phosphonamide **24c** to enone **118** afforded adduct **119** as a single isomer, with the attack occurring to the less hindered face of the enone. Further elaboration of the side chain was achieved by ozonolysis to give an aldehyde, selective reduction of the latter with 9-BBN, and protection of the obtained primary alcohol as its TBS ether. *N*-Deprotection to give **120** was followed by acylation with benzyloxalyl chloride and treatment with triethylphosphite at elevated temperature to yield tricyclic intermediate **121**. Cleavage of the TBS ethers and hydrogenolysis of the benzyl ester in presence of amidine **122** yielded trinem **23a** as its amidinium salt **123**.

Trinem **123** exhibited antibacterial activity against a variety of strains, with MIC’s of 1.0 μg/mL against *Staphylococcus aureus* 853E and 0.1 μg/mL against *Streptococcus pneumoniae* 3512. The antibacterial activity of **123** was considerably weaker compared to imipenem (**111**) and sanfetrinem (**113**), which showed MIC’s of 0.06 and 0.2 μg/mL, respectively, against *S. aureus* and 0.01 μg/mL against *S. pneumoniae*. The lack of potency of **123** was attributed in part to the missing α-orientated 4-alkoxy substituent present in sanfetrinem (**113**), which may act as a potential leaving group and appears to be crucial for activity [97].

#### Anthoplalone (1999)

**Scheme 15:**
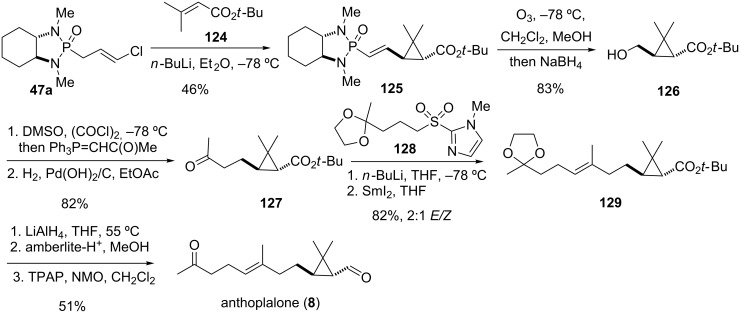
Total synthesis of (−)-anthoplalone (**8**) [56].

Isolated from the Okinawan actinian *Anthopleura pacifica,* anthoplalone (**8**) is a secosesquiterpene with a tetrasubstituted *trans*-cyclopropane subunit. The compound shows modest cytotoxic activity against murine melanoma cells [98–99]. The first enantioselective total synthesis of anthoplalone was achieved by Hanessian and co-workers and utilized their chloroallyl phosphonamide anion cyclopropanation methodology [56]. Thus, deprotonation of **47a** with butyllithium at low temperature and addition to *tert*-butyl 3,3-dimethylacrylate (**124**) provided adduct **125** as a single diastereomer (Scheme 15). Removal of the chiral auxiliary by ozonolysis and subsequent reduction afforded alcohol **126**. Chain extension was accomplished through a one-pot Swern oxidation/Wittig olefination protocol followed by hydrogenation to give ketone **127**. For further extension of the carbon chain and installation of the trisubstituted double bond, a modified Julia olefination with imidazole sulfone **128** was employed [100–102]. Thus, reaction of ketone **127** with the lithium anion of sulfone **128** and treatment of the obtained β-hydroxysulfone with SmI_2_ led to olefin **129** as a 2:1 mixture of *E/Z*-isomers. After reduction of the *tert*-butyl ester to the primary alcohol, the *E/Z*-isomers could be separated chromatographically. Cleavage of the ketal under acidic conditions to reveal the ketone moiety and final oxidation of the primary alcohol with tetrapropylammonium perruthenate (TPAP) completed the first enantioselective total synthesis of anthoplalone (**8**) and confirmed the absolute configuration of the natural product [56,103–104].

#### PTP inhibitors (2000)

Protein tyrosine phosphatases (PTPs) are part of a superfamily of enzymes that catalyze protein tyrosine dephosphorylation. They are key regulators in various, crucial kinase-dependent signal transduction pathways and act to counterbalance the kinases. In particular, PTP1B has attracted considerable attention for its role in the complex insulin-signaling pathway. It has been shown that overexpression of PTP1B contributes to diabetes and obesity [105–106]. Therefore, inhibititors of PTP1B may have potential as treatment for type-2 diabetes [107–110].

**Figure 7:**
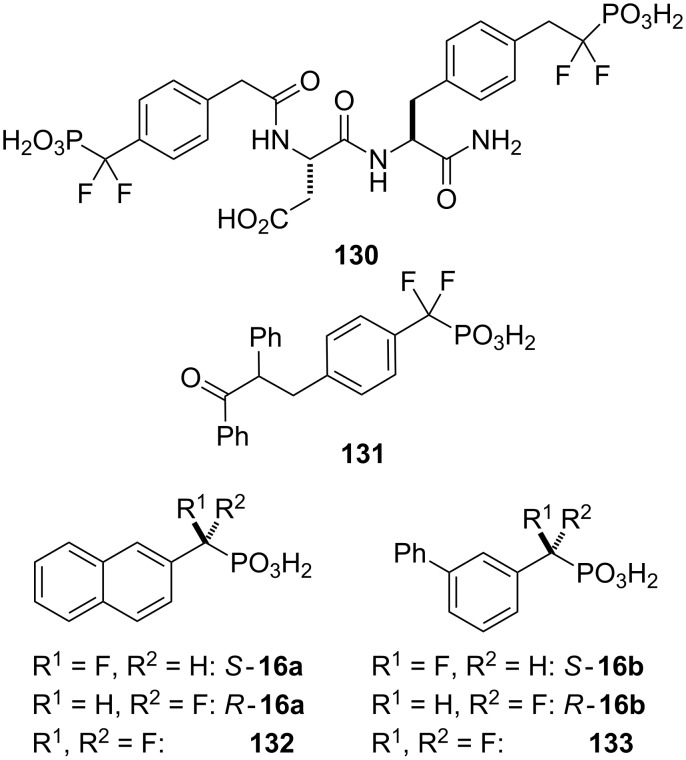
Protein tyrosine phosphatase (PTP) inhibitors **130**, **131** and model compounds **16**, **132** and **133** [68].

Hydrolytically-stable phosphotyrosyl mimetics have been developed as PTP1B inhibitors, including molecules such as **131** containing an α,α-difluoromethylenephosphonic (DFMP) moiety (Figure 7). In particular, peptides bearing a phosphonodifluoromethylphenylalanine (F_2_Pmp) group such as **130** have been shown to be among the most potent inhibitors with nanomolar potency against PTP1B [110–111].

**Scheme 16:**
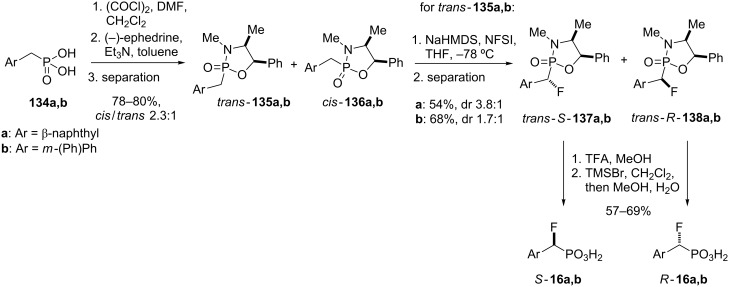
Synthesis of model PTP inhibitors **16a,b** [68].

Taylor and co-workers were interested to study α-monofluoroalkylphosphonic acids as PTP1B inhibitors in comparison to their difluoro analogues and compounds **16a,b** and **132**,**133** were chosen as model PTP1B inhibitors [68]. The enantiopure α-monofluoroalkylphosphonic acids were synthesized by diastereoselective fluorination of phosphonamides bearing (−)-ephedrine as chiral auxiliary, originally introduced by Sting and Steglich for the synthesis of aminoalkylphosphonic acids [7] (Scheme 16). Thus, condensation of the phosphonic acid dichloride obtained from **134a,b** with ephedrine yielded a separable mixture of diastereomeric phosphonamides, *trans*-**135a,b** and *cis*-**136a,b**. The fluorination with *N*-fluorobenzenesulfonimide (NFSI) of either *trans*-**135a** or *cis*-**136a** was found to be strongly dependant on the base used to generate the phosphonamidate anion. The best diastereomeric ratio of 3.8:1 (58% de) in favor of *trans*-*S*-**137a** was observed with NaHMDS as base in the reaction of *trans*-**135a**. The *cis*-isomer **136a** gave a similar result with NaHMDS, whereas the fluorination of *m*-(phenyl)benzyl phosphonamides **135b** and **136b** proved to be less selective. Although the diastereoselectivity was modest at best, the fluorinated products *trans*-*S*-**137a,b** and *trans*-*R*-**138a,b** could be readily separated by chromatography. Higher selectivities of up to 70% de were achieved in the fluorination step when *trans*-(*R*,*R*)-*N*,*N*’-dimethyl-1,2-diaminocyclohexane (**58**) was employed as chiral auxiliary, however, the diastereomeric products were not separable by chromatographic means.

Cleavage of the ephedrine auxiliary was accomplished by a three-step protocol. Treatment of **137a,b** and **138a,b** with trifluoroacetic acid in methanol followed by reaction with TMSBr and subsequent hydrolysis of the TMS ester gave the free acids (*S*)-**16a,b** and (*R*)-**16a,b**, respectively as pure enantiomers.

Compounds **16a,b**, **132** and **133** were found to be inhibitors of PTP1B. The monofluoro (*R*)-enantiomers *R*-**16a** (IC_50_ 675 µM) and *R*-**16b** (315 µM) were about 10-fold more potent than the corresponding (*S*)-enantiomers (IC_50_ 7500 µM and 3500 µM for *S*-**16a** and *S*-**16b**, respectively), but 10-fold less potent than the difluoro analogues **132** and **133** (IC_50_ 71 µM and 33 µM, respectively). The inhibition studies indicated that the pro-*S* fluorine in difluoro inhibitors **132** and **133** is essential for good inhibition, although the pro-*R* fluorine contributes significantly more towards PTP1B affinity [68].

#### MMP inhibitors (2001)

The matrix metalloproteinases (MMPs) are a family of structurally-related, zinc-containing enzymes that play a critical role in the degradation and remodelling of extracellular matrix. Over expression of MMPs has been associated with various physiological and pathological processes such as morphogenesis, angiogenesis, tissue repair, cirrhosis, arthritis, and metastasis, thus raising the possibility that inhibitors of these enzymes may possess therapeutic potential [112–113].

**Scheme 17:**
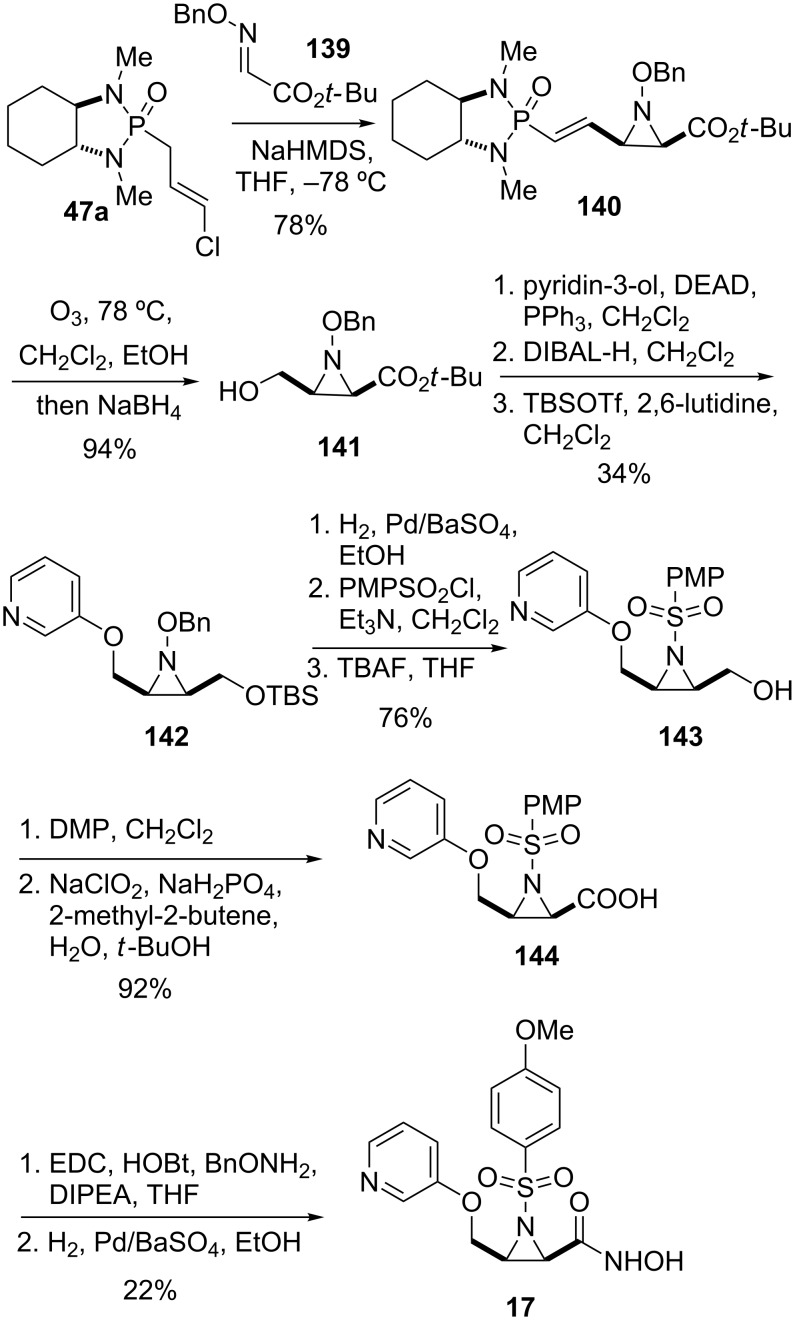
Synthesis of aziridine hydroxamic acid **17** as MMP inhibitor [63].

As part of studies on conformationally constrained MMP inhibitors by Hanessian and co-workers, *trans*- and *cis*-aziridines scaffolds were used as peptidomimetics to construct a series of hydroxamic acids analogs such as **17** (Scheme 17) [63]. While the *trans*-aziridines were prepared by conjugate addition of *O*-benzylhydroxylamine to α,β-unsaturated amides bearing a chiral oxazolidinone auxiliary, facile access to the *cis*-aziridine series was possible by using chiral chloroallyl phosphonamide **47a**. Thus, the addition of the anion of **47a** to *tert*-butylglyoxylate *O*-benzylamine (**139**) led to aziridine **140** as a single diastereomer. Ozonolysis followed by reductive work-up provided alcohol **141.** Coupling under Mitsunobu conditions with an appropriate alcohol, e.g., 3-hydroxypyridine, reduction of the *tert*-butyl ester with DIBAL-H, and treatment with TBS triflate gave silyl ether **142**. Hydrogenolysis of **142** using Pd/BaSO_4_ produced the free aziridine, which was then converted to the corresponding sulfonamide with *para*-methoxyphenyl (PMP) sulfonyl chloride. Cleavage of the silyl ether moiety with TBAF gave primary alcohol **143**, which was oxidized to the corresponding acid **144** by a two-step protocol consisting of treatment with Dess–Martin periodinane followed by Pinnick oxidation. Hydroxamic acid **17** was then obtained by coupling with *O*-benzylhydroxylamine followed by hydrogenolysis.

The *cis*-aziridine hydroxamic acid **17** showed good inhibitory activity against several matrix metalloproteinases, in particular MMP-3 and MMP-9, with IC_50_’s of 164 nM and 83 nM, respectively [63].

#### Methyl jasmonates and dihydrojasmonates (2001)

**Scheme 18:**
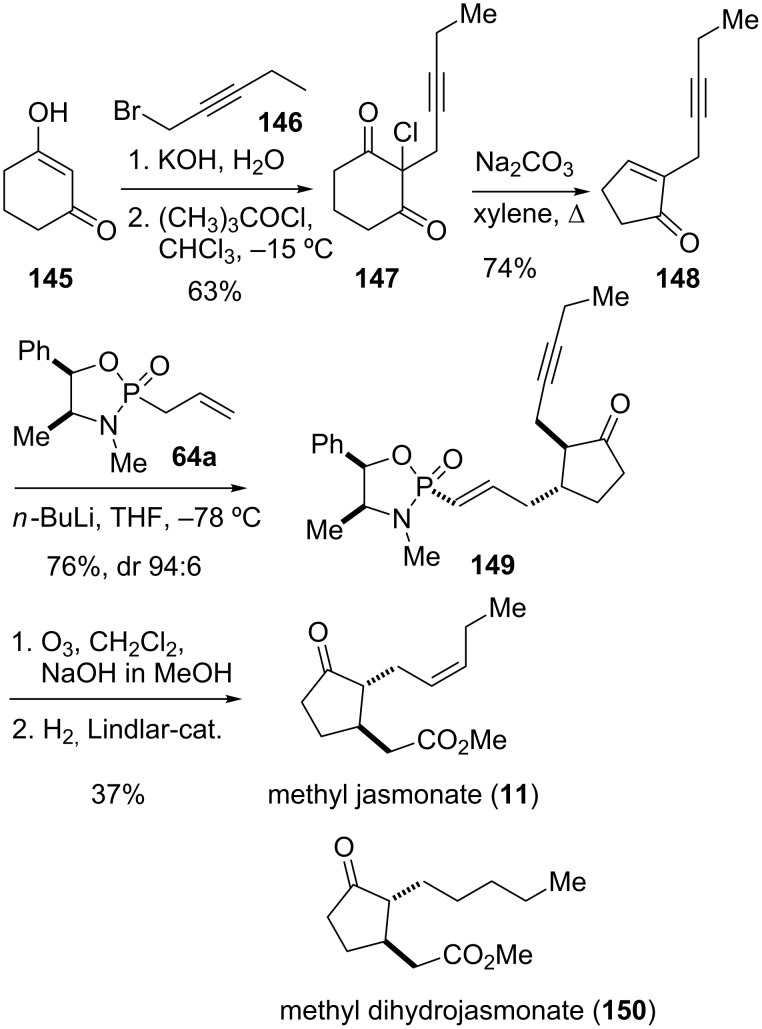
Synthesis of methyl jasmonate (**11**) [48].

The jasmonates, which comprise of methyl jasmonate (**11**) and the corresponding jasmonic acid, are important cellular regulators in plants. They participate in various developmental processes and defence mechanisms against biotic and abiotic stresses [114]. Originally isolated from *Jasminum grandiflorum*, the plant scent methyl jasmonate has found to be distributed ubiquitously in the plant kingdom. The unnatural analogue methyl dihydrojasmonate (**150**) possesses important olfactory properties and has become a major aroma chemical with a wide range of uses, mainly in fragrances (Scheme 18).

Hailes and co-workers were interested in developing a short synthetic route to both enantiomers of methyl jasmonate and methyl dihydrojasmonate, respectively [48]. To this end, they investigated the conjugate addition of chiral 2-propenylphosphonamides such as **64a**, derived from (1*R*,2*S*)-ephedrine, to α-substituted cyclopentenones. The required precursor for the synthesis of methyl jasmonate (**11**), 2-(2-pentynyl)-2-cyclopentene-1-one (**148**) was prepared by a known sequence [115] starting from 1,3-cyclohexanedione (**145**) (Scheme 18). Addition to 1-bromo-2-pentyne (**146**) followed by chlorination gave chlorodiketone **147**. The latter was then treated with sodium carbonate in boiling xylene to afford cyclopentenone **148**, presumably via decarbonylation of a cyclopropanone intermediate. Addition of the lithium anion of chiral phosphonamide **64a** at low temperature produced adduct **149** in good yield and diastereoselectivity. Cleavage of the phosphonamide auxiliary from **149** was achieved by ozonolysis in the presence of sodium hydroxide and methanol to give the corresponding methyl ester. The final reduction of the alkyne was carried out using the Lindlar catalyst to yield methyl jasmonate (**11**). Methyl dihydrojasmonate (**150**) was also synthesized using phosphonamide reagent **64a**, while replacing **148** with commercially available 2-pentyl-2-cyclopenten-1-one [48].

#### Nudiflosides A and D (2006)

**Figure 8:**
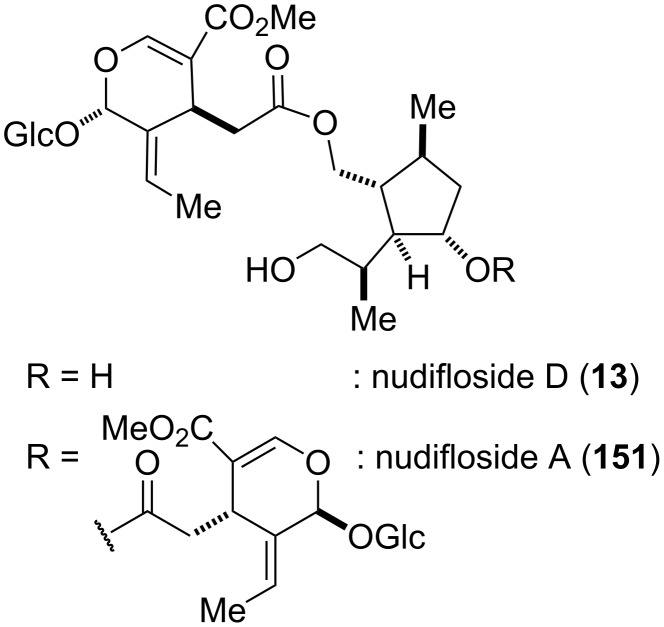
Structures of nudiflosides A (**137**) and D (**13**) [49].

**Scheme 19:**
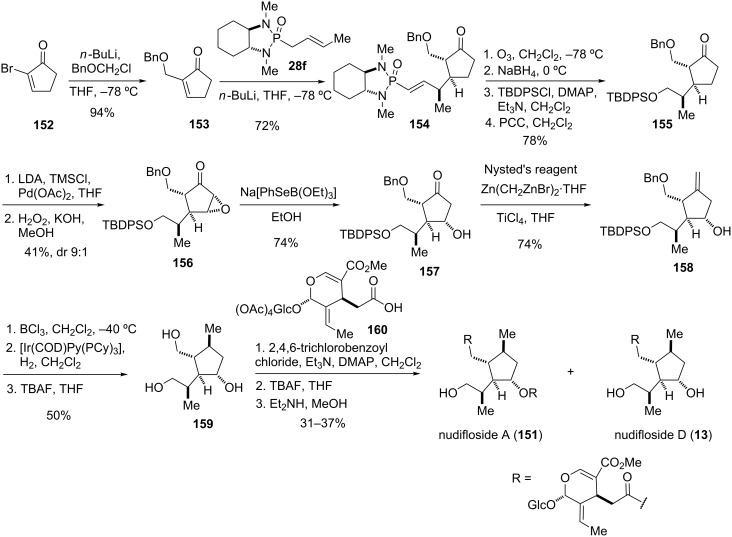
Total synthesis of the pentasubstituted cyclopentane core **159** of nudiflosides A (**151**) and D (**13**) and conversion to the natural products [49].

Extracts from *Jasminum nudiflorum* have been used as folk medicine in China for the treatment of inflammation and traumatic bleeding. The leaves and stems of this plant contain oleoside-type secoiridoid glucosides with structurally interesting tetrasubstituted cyclopentanoid monoterpene units [116]. Two representative examples of these glycosides are nudifloside A (**151**) and D (**13**), which share a common subunit (Figure 8) [116–118]. The first total synthesis of nudiflosides A and D was achieved by Hanessian and co-workers, which aimed at confirming their proposed structural and stereochemical assignment (Scheme 19) [49].

The correct installation of the stereocenters of the cyclopentane subunit **159** was dependent on the stereocontrolled Michael addition of the anion generated from crotyl phosphonamide **28f**, which set three contiguous stereocenters in one step. Thus, addition of the Li anion of **28f** to cyclopentenone **153** gave adduct **154** as a single diastereomer on a gram scale. Cleavage of the chiral auxiliary through ozonolysis followed by protection of the side chain as TBDPS ether afforded cyclopentanone **155**. Saegusa–Ito oxidation followed by epoxidation of the formed enone gave **156** as the major isomer (dr 9:1). Regioselective reductive opening of the epoxide with Na[PhSeB(OEt)_3_] produced hydroxy ketone **157**, which was then converted into the *exo*-methylene analogue **158** with Nysted’s reagent. Cleavage of the benzyl ether and stereocontrolled reduction of the olefin in the presence of Crabtree’s catalyst afforded a single isomer, presumably due to a directing effect of the adjacent hydroxy group. Final removal of the TBDPS protecting group gave cyclopentane triol **159**, which was esterified with varying equivalents of oleoside monomethyl ester peracetate **160** under Yamaguchi conditions [119–120] to give nudiflosides A (**151**) and D (**13**), respectively, thereby completing the synthesis and confirming the proposed stereochemistry [49].

#### Glutamate metabotropic receptor agonists (2000, 2007)

**Figure 9:**
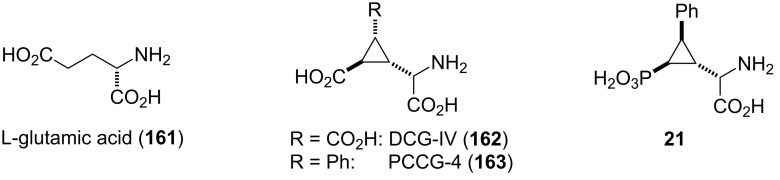
L-glutamic acid (**161**) and constrained analogues [57,124].

The metabotropic glutamate receptors (mGluRs) are members of the vast family of G-protein coupled receptors which are expressed throughout the central nervous system. They consist of at least eight sub-types, which are divided into three groups I–III. Through binding of glutamate **161** (Figure 9), the most abundant excitatory neurotransmitter in the mammalian central nervous system, the mGluRs are activiated and participate in the regulation of synaptic transmission and neuronal excitability through a metabotropic process. There is ongoing interest in mGluRs as drug targets, and the therapeutic potential of mGluR ligands for the treatment of CNS disorders and ailments such as Alzheimer’s and Parkinson’s disease, depression, anxiety, and schizophrenia is being validated [121–122].

**Scheme 20:**
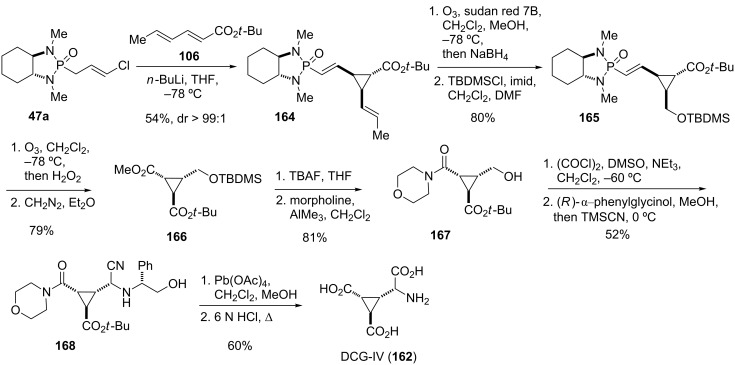
Stereoselective synthesis of DCG-IV (**162**) [57].

The discovery of group II mGluR agonist DCG-IV (**162**) (Figure 9) as a potent anticonvulsant and neuroprotective agent [123] had sparked interest in more efficient routes for its synthesis. Pellicciari and Marinozzi developed an asymmetric synthesis of DCG-IV (**162**) based on Hanessian’s cyclopropanation protocol (Scheme 20) [57]. Thus, addition of the Li anion generated from **47a** to *tert*-butyl sorbate (**106**) afforded the cyclopropane **164** as a single diastereomer. Selective ozonolysis of the propenyl side chain followed by reductive work-up and subsequent conversion of the formed primary alcohol into the corresponding TBDMS ether provided intermediate **165**. Removal of the chiral auxiliary and generation of the second carboxy moiety was then achieved by ozonolysis of **165** and ensuing esterification with diazomethane to give diacid ester **166**. Treatment of the latter with TBAF cleaved the TBDMS ether and gave a lactone intermediate, which was then opened by morpholine to afford amide **167**. The primary alcohol was then oxidized to the aldehyde under Swern conditions and submitted to a diastereoselective Strecker synthesis to install the amino acid moiety. Thus, condensation of the aldehyde with (*R*)-α-phenylglycinol followed by addition of trimethylsilylcyanide to the formed Schiff-base provided aminonitrile **168** as the major diastereomer (dr 95:5). Oxidative cleavage of the phenylglycinol moiety with Pb(OAc)_4_ liberated the amino-functionality and hydrolysis of the amide and nitrile under acidic conditions finally gave DCG-IV (**162**) [57].

**Scheme 21:**
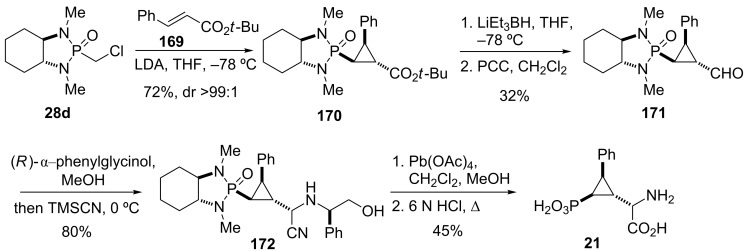
Stereoselective synthesis of mGluR agonist **21** [124].

Pellicciari and co-workers also reported on the synthesis of other constrained bioisosteres of L-glutamic acid, such as PCCG-4 (**163**) (Figure 9) [124]. To this end, phosphonocyclopropylamino acid **21** was designed as an analogue to **163** by replacing a carboxylic acid with a phosphonic acid moiety [60–61]. The stereoselective synthesis of **21** relied on another cyclopropanation protocol developed by Hanessian and co-workers [59]. Thus, conjugate addition of the anion of **28d** to (*E*)-*tert*-butyl cinnamate (**169**) proceeded with excellent stereocontrol, and adduct **170** was isolated as a single diastereomer (Scheme 21).

Conversion of *tert*-butyl ester **170** into aldehyde **171** by a two-step protocol was followed by condensation with (*R*)-α-phenylglycinol and treatment of the formed Schiff base with trimethylsilylcyanide to afford α-aminonitrile **172** as major isomer (dr > 4:1). Oxidative cleavage with Pb(OAc)_4_ liberated the amino functionality and hydrolysis of both the phosphonamide and nitrile groups under acidic conditions finally provided phosphonocyclopropylamino acid **21**. This compound showed to be a group III mGluRs selective ligand with moderate potency as mGluR4 and mGluR6 agonist (EC_50_ 59 µM and 51 µM, respectively) [60–61,124].

#### Berkelic acid (2009)

**Figure 10:**
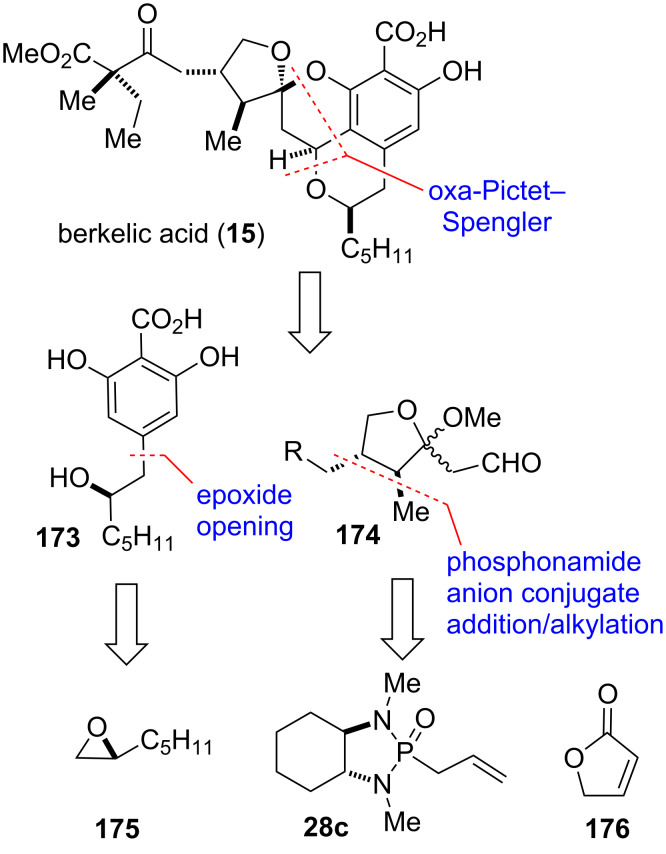
Key assembly strategy of berkelic acid (**15**) [43].

Berkelic acid (**15**) (Figure 10) is a spiroketal isolated from a fungus of the *Penicillium* species that grows in an unusual and harsh environment, Berkeley Pit Lake, an abandoned open-pit copper mine filled with acidic, metal-contaminated water [125]. The natural product shows moderate activity against MMP-3 and caspase-1, and high, selective activity toward ovarian cancer cell line OVCAR-3 with a GI_50_ of 91 nM. Both the relative configuration of the side chain as well as the absolute stereochemistry of the molecule was originally not assigned. The interesting biological profile in combination with the unknown stereochemical assignments made berkelic acid an attractive target for total synthesis, with the first one completed by Snider and co-workers [43].

The tetracyclic core of berkelic acid (**15**) was thought to be assembled through an oxa-Pictet–Spengler reaction from 2,6-dihydroxybenzoic acid **173** and ketal aldehyde **174** as key building blocks (Figure 10). The 2,6-dihydroxybenzoic acid **173** is accessible by opening of epoxide **175** as chiron with a suitable nucleophile. The ketal aldehyde **174** would be derived from butenolide **176** through a conjugate addition sequence employing phosphonamide **28c**, thereby setting two of the three stereocenters of the five-membered ring in a single step.

**Scheme 22:**
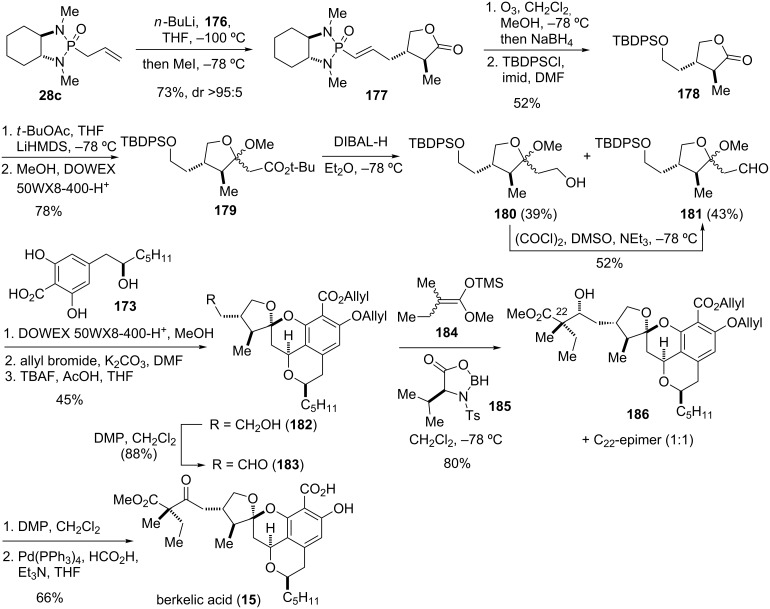
Total synthesis of berkelic acid (**15**) [43].

Thus, deprotonation of chiral phosphonamide **28c** and addition of the anion to 2(5*H*)-furanone (**176**) at −100 ºC, followed by trapping with excess methyl iodide afforded adduct **177** with excellent selectivity (dr > 95:5) (Scheme 22). Ozonolysis with reductive work-up and ensuing protection of the formed hydroxy group as TPDPS ether provided lactone **178**. Addition of the enolate of *tert*-butyl acetate to **178** and ketal formation afforded **179**. Reduction with DIBAL-H gave ketal aldehyde **181**, which was then condensed with 2,6-dihydroxybenzoic acid **173** in a oxa-Pictet–Spengler reaction to form the tetracyclic core of berkelic acid. Treatment of the obtained tetracyclic salicylic acid with allyl bromide and desilyation with TBAF/AcOH provided **182**. The primary alcohol of the side chain was oxidized with Dess–Martin periodinane (DMP) to give aldehyde **183**. The latter was subsequently reacted with trimethylsilyl ketene acetal **184** in the presence of oxazaborolidinone **185** to afford aldol product **186** and the C_22_-epimer as only isomers in a 1:1 mixture. Dess–Martin oxidation and deprotection of both allyl groups with formic acid under palladium catalysis finally provided berkelic acid (**15**). Thus, total synthesis of both epimers established the relative configuration of the side chain at C_22_ which was previously unknown, as well as helped to determine the absolute stereochemistry of the molecule [43,126–132].

#### Ambruticin S and jerangolid A (2010)

**Figure 11:**
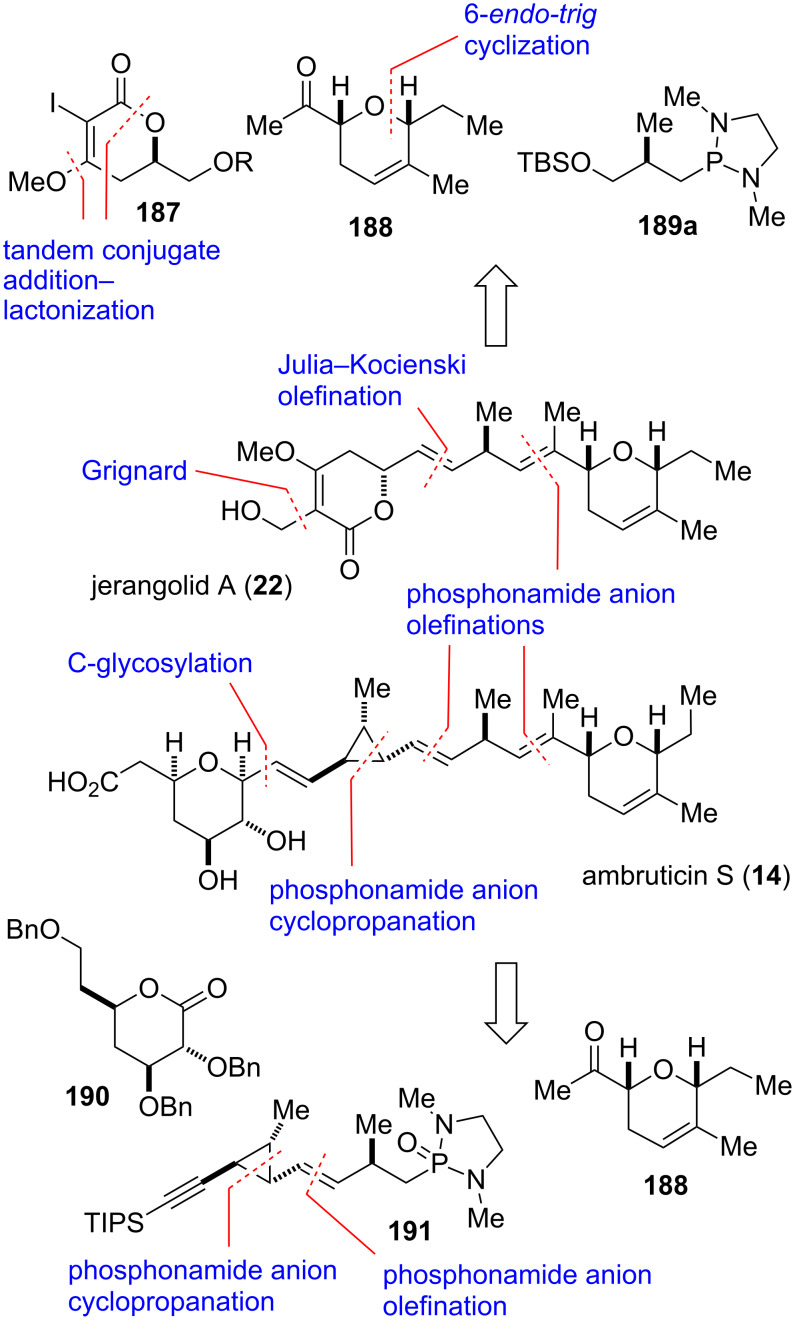
Key assembly strategy of jerangolid A (**22**) and ambruticin S (**14**) [27–28].

The jerangolids [133–134] and the ambruticins [135–137] are part of two closely related families of linear polyketides with potent antifungal properties produced by a variety of myxobacteria. Besides the biochemical profile, the two families share common structural features and a common biosynthesis [138–139]. Among the five members of the jerangolid family, which may be considered as trunctated analogs of the ambruticins, jerangolid A is reported to be the most potent [133–134]. The ambruticin family currently consists of eight known members [140]. Since the “eastern” segment of jerangolid A (**22**) and ambruticin S (**14**) is identical, a synthetic strategy was considered for this segment that would allow for the total synthesis of both molecules (Figure 11) [27–28].

The strategy for the first synthesis of jerangolid A (**22**) is depicted in retrosynthetic format featuring dihydropyran **188**, lactone **187**, and phosphonamide **189a** originating from the Roche ester (Figure 11) [27]. Lactone **187** ring was thought to be formed from addition of ethyl propiolate to (*S*)-glycidol, and ensuing conjugate addition of methanol and lactonization. The second cyclic building block, *syn*-dihydropyran **188**, could be synthesized by a highly diastereoselective 6-*endo-trig* cyclization of an allylic 1,3-diol, which was developed by Hanessian and co-workers [141]. The required allylic 1,3-diol would also be accessed from (*S*)-glycidol. The assembly of the two cyclic building blocks and the Roche ester derived middle fragment under formation of the *trans*-double bonds would utilize phosphonamide and sulfone anion coupling strategy, respectively. A similar strategy was employed for the assembly of ambruticin S (**14**). Disconnection at logical sites led to lactone **190** derived from D-glucose, dihydropyran methyl ketone **188**, and phosphonamide **191** as advanced intermediates [28]. Building block **191** and three of its four stereocenters would be constructed via phosphonamide-mediated olefination and cyclopropanation reactions [51–55]. The remaining stereocenter would originate from Roche ester as a readily available chiron.

**Scheme 23:**
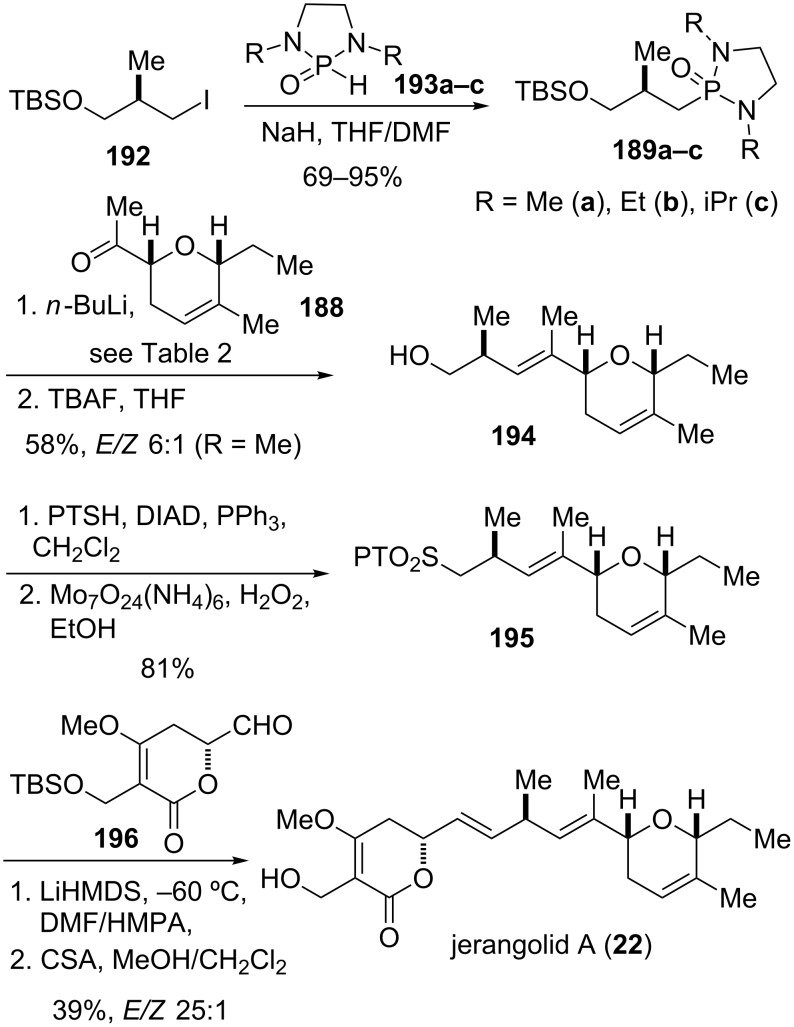
Final assembly steps in the total synthesis of jerangolid A [27].

The final steps in the assembly of jerangolid A (**22**) are shown in Scheme 23. The required cyclic phosphonamide reagent **189** for the olefination of methyl ketone **188** was obtained from alkylation of 1,3-dialkyl-2-oxo-1,3,2-diazaphospholidines **193a–c** [69–71] with iodide **192**. The latter was prepared from (*S*)-Roche ester in a three step sequence. Coupling of methyl ketone **188** with phosphonamides **189a–c** afforded separable mixtures of *E/Z* isomers of TBS ether **197**, gratifyingly with the desired *E*-isomer as the major product (Table 2).

**Table 2:** Olefination of ketone **188** employing cyclic phosphonamides **189** [27].^a^

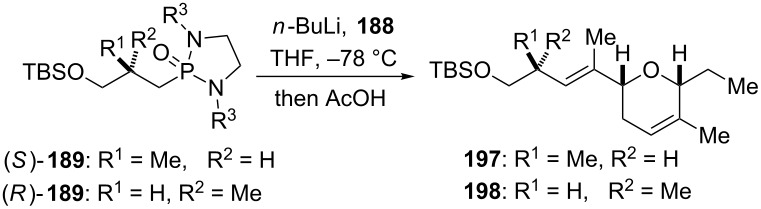				

Entry	Phosphonamide	Product	Yield (%)	*E*/*Z*

**1**	(*R*)-**189a**: R^3^ = Me	**197**	57	3:1
**2**	(*R*)-**189a**: R^3^ = Me	**197**	62	6:1^b^
**3**	(*R*)-**189b**: R^3^ = Et	**197**	38	5:1
**4**	(*R*)-**189c**: R^3^ = iPr	**197**	20	13:1
**5**	(*S*)-**189a**: R^3^ = Me	**198**	63	19:1

^a^Reaction conditions: **189**, *n*-BuLi, THF, −78 °C, 1 h; **188**, −78 °C, 1 h; then AcOH (xs), −78 °C to rt. ^b^Addition of AcOH at rt.

The reaction conditions, the steric nature of the phosphonamide *N*-substituents as well as its absolute configuration had a significant impact on the stereoselectivity of the olefination. Thus, treatment of methyl ketone **188** with dimethyl phosphonamide **189a** afforded the corresponding olefin **197** in a 3:1 *E*/*Z* ratio (Table 2, entry 1). Equilibrating the reaction mixture at ambient temperature before adding acetic acid helped to improve the *E*/*Z* ratio to 6:1 (Table 2, entry 2). The *E*/*Z* selectivity could further be enhanced by increasing the steric demand of the phosphonamide substituents. Replacing methyl with isopropyl improved the selectivity from 3:1 to 13:1, albeit to the expense of lower conversion and increased recovery of starting material (Table 2, entry 1 and 4). Remarkably, the (*S*)-enantiomer of dimethyl phosphonamide **189a** furnished an excellent *E*/*Z* ratio of 19:1 in the olefination of **188** to afford diastereomer **198** (Table 2, entry 5).

Deprotection of TBS ether **197** with TBAF provided alcohol **194** which was then transformed into known phenyltetrazole (PT) sulfone **195** [142–143] through Mitsunobu reaction with 1-phenyltetrazole-5-thiol (PTSH), followed by oxidation of the intermediate sulfide. Coupling of the lactone building block in form of aldehyde **196** with the fully elaborated PT-sulfone **195** provided the corresponding olefin with the correct double-bond geometry in moderate yield and excellent selectivity (*E*/*Z* > 25:1). Lastly, cleavage of the TBS ether under mild acidic conditions afforded jerangolid A (**22**) [27,143–144].

**Scheme 24:**
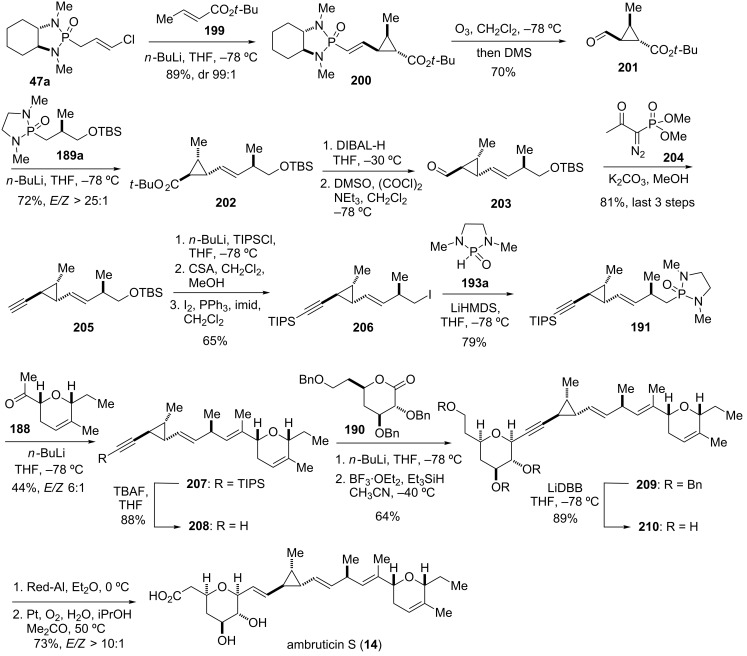
Key assembly steps in the total synthesis of ambruticin S (**14**) [28].

The synthesis of ambruticin S commenced with the 1,4-conjugate addition of the anion of chiral *trans*-chlorallyl phosphonamide *ent*-**47a** to *tert*-butyl crotonate (**199**) to give cyclopropane **200** as a single diastereomer with the desired relative and absolute stereochemistry (Scheme 24) [28]. Removal of the chiral auxiliary by oxidative cleavage of the olefin furnished aldehyde **201**, which was coupled with phosphonamide **189a** to afford olefin **202** in good yield and excellent selectivity (*E/Z* > 25:1). The latter was then converted into alkyne **205** via DIBAL-H reduction of the *tert*-butyl ester moiety followed by Swern oxidation to give aldehyde **203**, and treatment with the Ohira–Bestmann reagent **204** [145–146]. Protection of the alkyne CH as its TIPS-derivative, chemoselective removal of the TBS group using CSA and transformation of the obtained primary alcohol with iodine and PPh_3_ gave iodide **206**. The latter was then converted into phosphonamide **191** by treatment with the lithium anion of 1,3-dimethyl-2-oxo-1,3,2-diazaphospholidine (**193a**). Coupling with methyl ketone **188** was performed in a similar fashion as described for jerangolid A. Thus, deprotonation of phosphonamide **191** with *n*-butyllihium and treatment with methyl ketone **188** followed by addition of acetic acid provided triene **207** as the desired major isomer with moderate selectivity (*E*/*Z* 6:1). Treatment of **207** with TBAF liberated alkyne **208**, which was coupled with lactone **190** in a two-step sequence to give the desired *syn*-dihydrofuran **209** as a single diastereomer. Next, cleavage of the three benzyl ether moieties without reducing any of the double-bonds was achieved using lithium 4,4-di-*tert*-butylbiphenylide (LiDBB) to deliver alkyne **210**. The homopropargylic system was reduced with sodium bis(2-methoxyethoxy)aluminum hydride to afford the corresponding olefin with good selectivity (*E*/*Z* > 10:1). Finally, selective oxidation of the primary hydroxy group was achieved using a method that was chosen before by Jacobsen for the same transformation [147]. Thus, treatment of the intermediate triol with oxygen under platinum catalysis efficiently oxidized the primary alcohol group to the carboxylic acid without affecting the two secondary hydroxy groups and provided (+)-ambruticin S (**14**) [28,142,147–151].

#### Estrone (2010)

Estrone (**12**), an aromatized C18 steroid with a 3-hydroxy group and a 17-ketone, is a member of the estrogenic hormones, which also include estriol and estradiol. In humans, it is produced primarily by the cyclic ovaries, placenta, and the adipose tissue of men and postmenopausal women [152].

**Figure 12:**
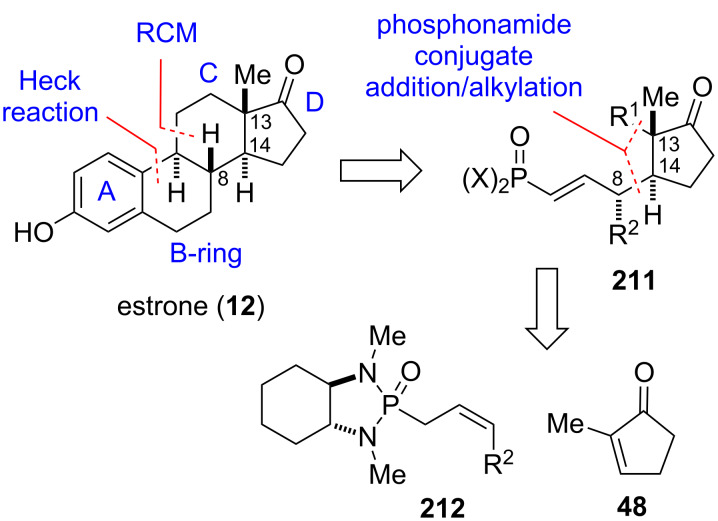
General steroid construction strategy based on conjugate addition of **212** to cyclopentenone **48**, exemplified with estrone (**12**) [44].

A classic problem in steroid synthesis is the selective formation of the *trans*-fused ring junction. A common strategy to circumvent this issue is to employ a cyclization strategy that commences from a D-ring precursor already containing the correct stereochemistry at the future CD ring junction [153–154]. Linclau and co-workers developed this concept further and a general steroid construction strategy based on the formation of a D-ring template that contains the correct configuration of three stereocenters C8, C13 and C14 with suitable functionalization for the following C- and B-ring cyclizations (Figure 12). The applicability of this approach to steroid synthesis was validated using estrone as target [44]. A key requirement for the strategy was a highly diastereo- and enantioselective formation of the D-ring intermediate **211** and flexibility in introducing different R^1^ and R^2^ groups to enable the synthesis of diverse steroids targets. Intermediate **211** was envisioned to come from a one-pot process involving the conjugate addition of a chiral phosphonamide anion to cyclopentenone **48** followed by an alkylation to introduce R^1^. Furthermore, the obtained vinylic phosphonamide was thought to be an excellent reactive handle for the following C-ring cyclization.

**Scheme 25:**
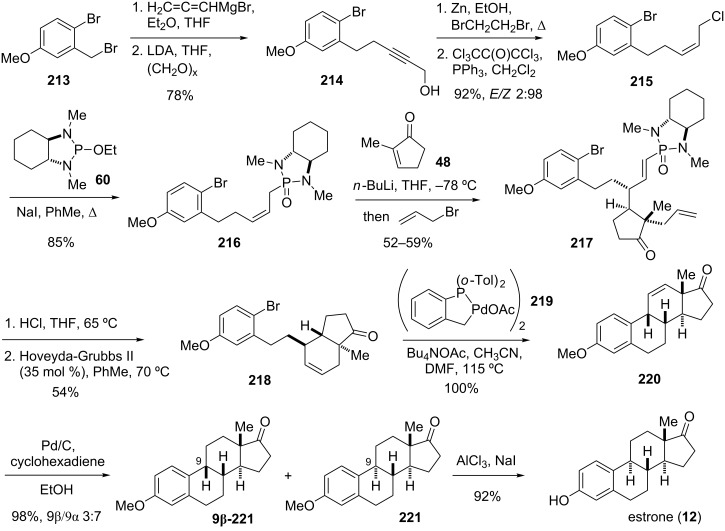
Total synthesis of estrone (**12**) [44].

The synthesis of the required *Z*-allylic phosphonamide **216** began from dibromide **213** by benzylic displacement with allenylmagnesium bromide followed by reaction with paraformaldehyde to give propargylic alcohol **214** (Scheme 25). Alkyne reduction was performed with Zn and dibromoethane to give selectively the corresponding *cis*-alkene. The Zn/dibromoethane system proved to be more reliable for this reduction than using hydrogen and poisoned Pd-catalysts. Chlorination with hexachloroacetone gave allylic chloride **215**, which was then converted to phosphonamide **216** via an Arbuzov reaction with phospholane **60**. Deprotonation of **216** and addition to cyclopentenone **48** followed by alkylation with allyl bromide afforded adduct **217** as a single diastereomer.

With key intermediate **217** in hand, the B and C ring systems were then constructed by two subsequent cyclizations. Thus, treatment of the phosphonic acid obtained from acid hydrolysis of **217** with Hoveyda–Grubbs II catalyst afforded *trans*-hydrindene **218**, with the Δ9,11 double bond perfectly positioned for the subsequent Heck B-ring closure. Cyclization product **220** was then obtained in quantitative yield by treatment of **218** with catalytic amounts of palladacyle **219** at elevated temperature, albeit with the undesired 9β-configuration. Inversion of the C9 stereocenter and reduction of **220** was achieved by an isomerization/hydrogenation process using Pd/C and cyclohexadiene, which produced **221** and its C9 epimer **9β-221** in a 7:3 mixture. Separation of the desired isomer by crystallization and cleavage of the methyl ether finally gave estrone (**12**). Three of the four stereocenters of estrone [155–163] were set in a single conjugate addition and enolate alkylation reaction with excellent stereocontrol [44].

## Conclusion

In this review we summarized a substantial volume of work dealing with the preparation, reactivity, and utility of cyclic phosphonamides as versatile reagents for the asymmetric synthesis of a variety of acyclic and carbocyclic chiral non-racemic compounds. The focus was placed on enantiomerically pure pentacovalent *C_2_*-symmetrical phosphonamides, whose stabilized anions have been used as nucleophilic reagents toward electrophiles in a variety of applications. Especially useful among others are tandem conjugate additions to α,β-unsaturated carbonyl substrates followed by alkylation of the resulting enolates to give diastereomerically enriched acyclic, carbocyclic, and azacyclic molecules harboring as many as three contiguous stereogenic centers. These have been useful starting materials for the synthesis of a number of natural products and biologically active molecules such as enzyme inhibitors, bioisosteres, and receptor agonists to mention a few. Although the initial products must be oxidatively cleaved to obtain the corresponding aldehydes, thereby sacrificing the original phosphonamide portion, the benefits are in the highly functionalized products that are obtained, many of which are not easily attainable by other means. The methods are also of great utility for the stereocontrolled synthesis of the medicinally important α-substituted phosphonic acids in the case of alkyl halides as electrophiles. The utility of chiral non-racemic phosphonamides in organic synthesis extends beyond their uses as mild carbon-based nucleophilic reagents for stereoselective alkylation, amination, Michael addition, cyclopropanation and aziridination reactions.

For example, diastereoselective *o*-metalation of ferrocenes was mediated by (*R*,*R*)-*N*,*N*’-dimethyl-1,2-diaminocyclohexane [164]. 2-Dimethylamino-*N,N’*-diphenyl-1,3,2-diazaphospholidine is an excellent reagent for the conversion of alcohols to the corresponding crystalline 2*-*alkoxy*-N*,*N*’-diphenyl-1,3,2-diazophospholidines, simply by heating in toluene with elimination of dimethylamine [165]. The resulting products are excellent substrates for Arbuzov-type S_N_2 halogenations with methyl iodide or bromine as halogen sources. Related *C*_2_-symmetrical diazaphospholidines can be used for the determination of enantiomeric excesses of chiral alcohols by ^1^H NMR [166] and carboxylic acids [167].

The possibility to perform ring-closing metathesis reactions with α,β-unsaturated phosphonic acids resulting from the hydrolysis of the initially formed phosphonamide, as in the recent synthesis of estrone [44], adds a new and exciting dimension to the utility of phosphonamides in asymmetric synthesis.
